# Frequency Stability Degradation in Quartz and MEMS Oscillators

**DOI:** 10.3390/s26175425

**Published:** 2026-08-27

**Authors:** Mariusz Mścichowski, Paweł Kwiatkowski, Klaudia Majchrowicz, Ryszard Szplet

**Affiliations:** 1 Institute of Optoelectronics, Military University of Technology, gen. Sylwestra Kaliskiego 2, 00-908 Warsaw, Poland; 2 Faculty of Electronics, Military University of Technology, gen. Sylwestra Kaliskiego 2, 00-908 Warsaw, Poland

**Keywords:** quartz oscillators, TCXO, OCXO, MEMS oscillators, frequency drift, temperature, aging, vibration, acceleration sensitivity, hydrostatic pressure

## Abstract

The operating frequency of a resonant oscillator is determined by the coupled behavior of the resonator, sustaining electronics, package, power supply, mounting conditions, and operating environment. Frequency stability is critical in sensor systems, where oscillators provide references for sampling, synchronization, phase-sensitive measurements, sensor fusion, and distributed sensing. This review examines how these factors affect the short- and long-term stability of quartz oscillators (XOs, TCXOs, and OCXOs) and microelectromechanical systems (MEMS) oscillators. Organized by physical cause rather than device type, the review covers temperature, including gradients and hysteresis, aging, acceleration, vibration, shock, pressure, humidity, power-supply and load variations, electric and magnetic fields, electromagnetic interference, and ionizing radiation. For each factor, the dominant degradation mechanisms and compensation methods are compared across both technologies. The comparison indicates that OCXOs retain an advantage in low-noise timekeeping over long averaging times, whereas advanced MEMS oscillators can approach quartz performance in selected operating regimes while offering smaller size, monolithic integration, and, in ruggedized products, 0.01 ppb/g acceleration sensitivity and 20,000 g shock ratings. In the literature reviewed, appropriate measurement methods exist but are applied inconsistently across technologies. Oscillator selection should therefore be based on a comprehensive error budget covering all relevant environmental and system-level factors.

## 1. Introduction

**Figure 1:**
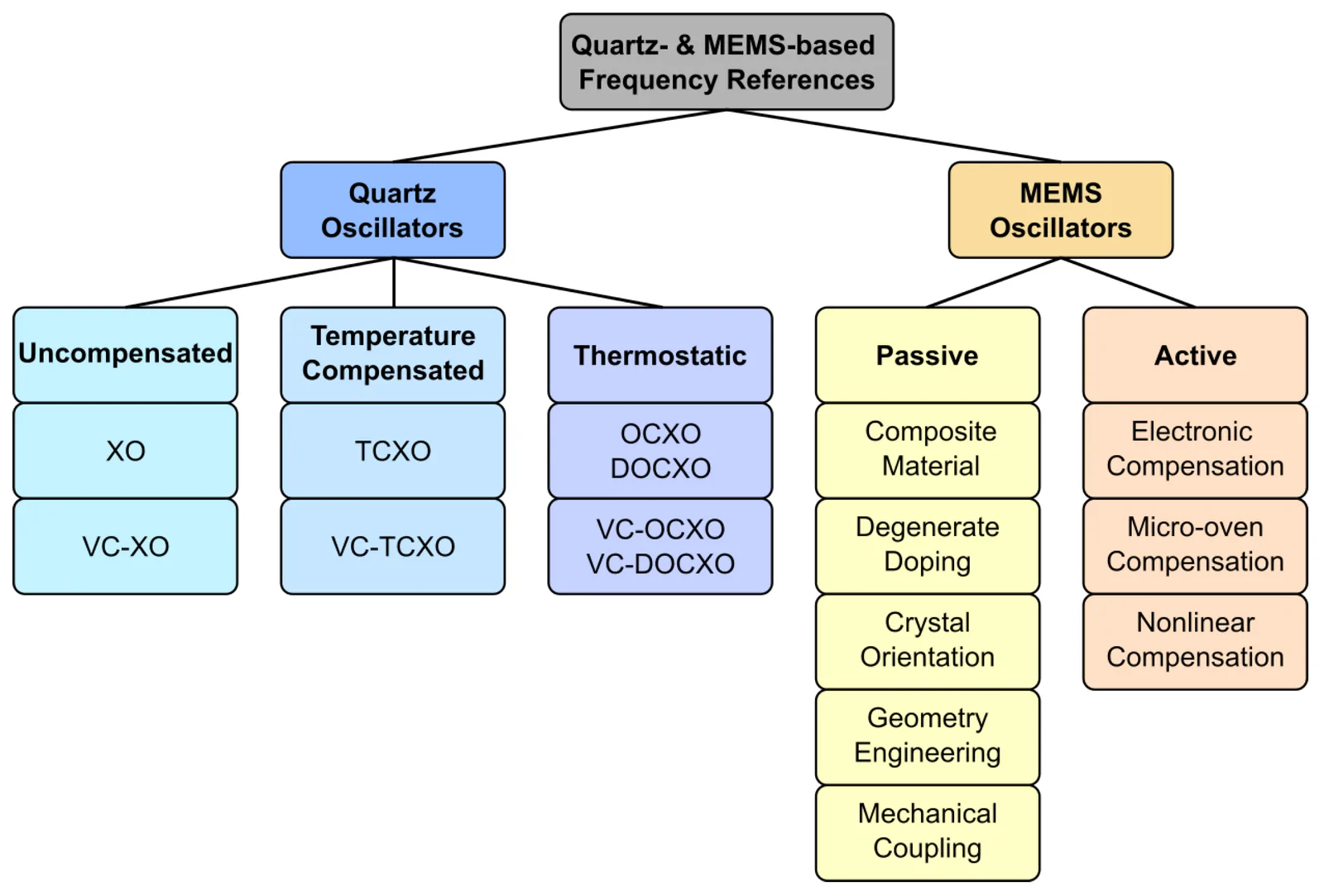
Classification of quartz and MEMS oscillators according to the methods used to improve frequency stability.

A reference frequency source is a fundamental component of many timing and synchronization systems. Stable local oscillators are essential in wireless sensor networks and Internet of Things (IoT) devices, where they affect synchronization accuracy among distributed nodes [1]. In telecommunication networks, oscillator instability contributes to phase and time errors, and synchronization requirements are specified in ITU-T recommendations, for example for 5G networks [2]. In GNSS receivers, the local clock is used in receiver time estimation and affects the behavior of the solution under weak signal conditions and during motion [3,4]. In the power grid, timing accuracy determines the consistency of measurements and the analysis of grid state [5,6], while in data centers precise synchronization enables correct ordering of events in distributed systems [7]. Frequency instability in software-defined radio (SDR) platforms directly affects the local-oscillator error and the measured baseband frequency, which is particularly important in frequency-measurement-based applications such as Doppler-based localization [8] and SDR-based sensing [9,10]. If the oscillator frequency shifts even by a small amount, the phase error accumulates over time. For this reason, it is necessary to assess not just a single datasheet number but also the target operating conditions such as temperature, hysteresis, aging, warm-up time, airflow, vibration, pressure, power supply, and electrical load [11,12,13]. The operating frequency of resonant oscillators should be treated as a dynamic operating point of an electromechanical system in which the resonator provides the frequency-selective element, while the actual frequency also depends on the sustaining electronics, packaging, mounting method, power supply, load, and operating environment. An oscillator described in a datasheet by a single nominal frequency may behave differently in the target device following temperature variations, after a heating and cooling cycle, under vibration, after several months of aging, or even after being soldered to a board with a different distribution of stresses and parasitic capacitances [13,14,15]. In an uncompensated quartz crystal oscillator (XO), the frequency reference is a quartz resonator that is not actively isolated or compensated against many environmental variables, therefore it is used as the reference case for analyzing how external variables affect oscillator stability. Quartz oscillators also have a well-established classification based on the method of temperature compensation, as shown in Figure 1.

In a temperature-compensated crystal oscillator (TCXO), temperature sensing and correction are added to the basic XO structure, most often by tuning a varactor diode or by digitally switching capacitive elements. In an oven-controlled crystal oscillator (OCXO), the crystal is instead held at an approximately constant internal temperature above the ambient temperature [14,15,16]. In a double oven-controlled crystal oscillator (DOCXO), the inner crystal oven is enclosed within a second thermal enclosure, which further improves temperature stability. Each of these oscillator types can also include a voltage-control (VC) input, typically used for electronic frequency correction or disciplining by an external control loop. For clarity, voltage-controlled variants are denoted here using the prefix VC-, namely VC-XO, VC-TCXO, VC-OCXO, and VC-DOCXO.

Microelectromechanical-system (MEMS) oscillators, despite serving the same purpose as frequency references, differ fundamentally in their construction. A MEMS oscillator is based on a micromechanical structure, usually made of silicon or a piezoelectric material [17]. It is most often combined with an integrated circuit (IC) that amplifies, corrects, and multiplies the signal received from the micromechanical structure and enables frequency trimming and compensation. The two technologies are usually described at different levels of abstraction, and Figure 1 separates these levels explicitly. Quartz devices are classified at the system level, because XO, TCXO, OCXO, and DOCXO are complete oscillator architectures, each defined by the way temperature is handled outside the resonator. MEMS devices are classified at the resonator and circuit level, because there the compensation mechanism is embedded in the material, geometry, and vibration mode of the resonator itself, or added by the co-integrated electronics; passive methods act before any control loop is applied, whereas active methods require sensing and feedback. In this article, MEMS oscillators are treated as a separate technology competing with quartz oscillators in terms of stability, mechanical robustness, size, power, and integration capability [18,19,20].

**Figure 2:**
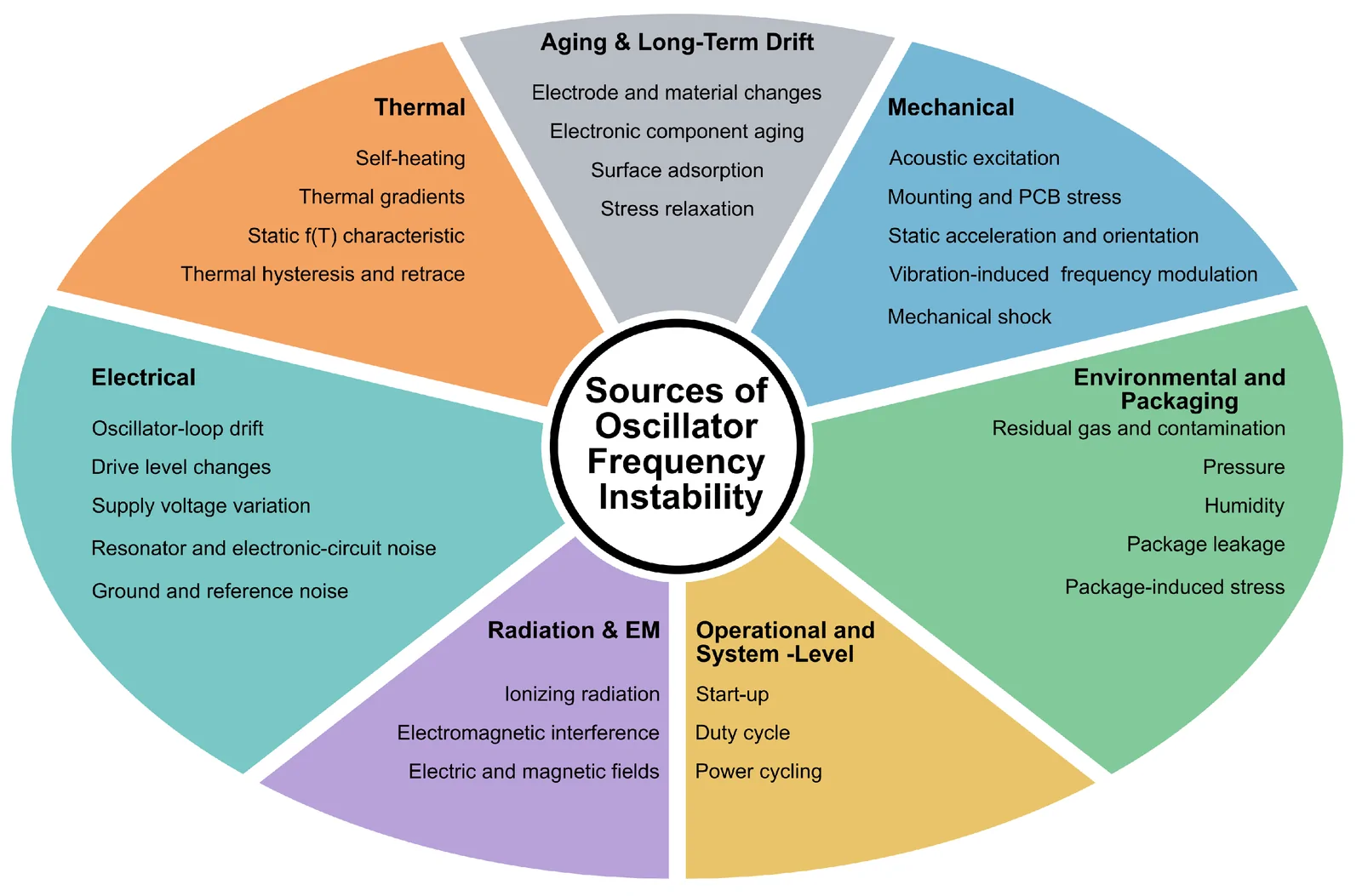
Classification of the main factors contributing to short- and long-term frequency instability in quartz and MEMS oscillators.

The frequency stability of quartz and MEMS oscillators is affected by a broad range of thermal, mechanical, electrical, environmental, packaging-related, and operational factors. These mechanisms may influence the resonator directly or act indirectly through the oscillation circuit, package, mounting, and surrounding system, often interacting with one another. This is particularly important in sensor systems, where oscillators provide the timing and frequency references required for sampling, synchronization, phase-sensitive measurements, sensor fusion, and communication between distributed nodes. Frequency instability can therefore propagate into timing errors, measurement drift, phase uncertainty, and reduced coherence between sensors. To support a comprehensive comparison of short- and long-term stability degradation in quartz and MEMS technologies, the main sources of frequency variation, drift, and instability are summarized in Figure 2. Particular attention is given to environmental effects and to the compensation and mitigation methods discussed in the following sections.

Existing reviews emphasize MEMS resonator technology [17,21], oven-controlled MEMS architectures [22], or temperature-coefficient compensation [18], while general frequency-stability reviews typically address a single metric or technology [23,24] rather than a unified cross-technology causal framework. The added value of this review is a common cause-based framework for quartz and MEMS devices that connects mechanisms originating in the resonator, package, electronics, and operating environment with sensor-system errors and the construction of an application-specific error budget. Throughout the comparison, the evaluation level of each result is identified, and the reported numerical values are retained with their test conditions. Appendix A collects the results that can be compared within a stated metric and set of conditions.

## 2. Measures of Frequency Stability

### 2.1. Terminology and Definitions

Frequency accuracy, frequency stability, drift, aging, phase noise, and jitter describe different aspects of oscillator performance and should not be used interchangeably. Frequency accuracy describes the closeness of the measured or mean oscillator frequency to the nominal frequency $f_{0}$ or to a reference frequency under specified operating and measurement conditions. Frequency stability describes the variation of oscillator frequency over time for a specified observation or averaging interval. Consequently, an oscillator may exhibit high frequency stability while having poor frequency accuracy if its frequency remains nearly constant but is offset from $f_{0}$. Conversely, its mean frequency may be close to $f_{0}$, while temporal fluctuations result in poor frequency stability [11,12].

The basic normalized quantity used to describe oscillator frequency error is the fractional frequency deviation, which defines the instantaneous frequency offset as the ratio of the difference between the actual frequency f(t) and the nominal frequency $f_{0}$ to the nominal value [11,12]:

(1) $$y\left(t\right)=\frac{f\left(t\right)-f_{0}}{f_{0}}=\frac{\Delta f(t)}{f_{0}}.$$

This normalization allows oscillators with different nominal frequencies to be compared and their errors to be expressed in ppm (parts per million, $10^{-6}$), ppb (parts per billion, $10^{-9}$), or ppt (parts per trillion, $10^{-12}$) [11,12].

In this review, drift is defined as a slow, systematic frequency change caused by internal or external factors, including changes in temperature, supply voltage, load, stress, or electronic parameters. Aging is used more narrowly for the long-term frequency change caused by time-dependent physical and chemical processes within the resonator, package, mounting structure, and sustaining electronics under nominally constant environmental and operating conditions. Thus, aging represents one specific source of long-term frequency drift and should be distinguished from drift caused by changes in environmental or operating conditions. Phase noise and jitter are complementary descriptions of short-term phase fluctuations. Phase noise represents these fluctuations in the frequency domain, whereas jitter describes the corresponding variation of clock edge or zero crossing times in the time domain. When jitter is inferred from integrated phase noise data, the numerical relationship depends on the carrier frequency and on the phase-noise integration bandwidth. More generally, a reported jitter value should be accompanied by its definition, observation interval, and measurement bandwidth [11,25,26]. Table 1 summarizes the terminology used throughout this review.

### 2.2. Time-Domain and Frequency-Domain Measures

Time-domain frequency stability is commonly quantified using Allan variance and related statistics. Allan introduced the two-sample variance (AVAR) as a statistical measure of frequency-standard instability [29,30]. Allan deviation (ADEV) is the square root of that variance and quantifies the mean squared difference between consecutive fractional-frequency averages computed over an averaging time τ:

(2) $$\sigma_{y}\left(\tau \right)=\sqrt{\frac{1}{2}\langle {\left({\bar{y}}_{k+1}\left(\tau \right)-{\bar{y}}_{k}\left(\tau \right)\right)}^{2}\rangle },$$

where ${\bar{y}}_{k}\left(\tau \right)$ is the average fractional frequency deviation over the *k*-th time interval of duration τ, and 〈·〉 denotes averaging over all adjacent interval pairs. For a finite sequence of *M* fractional-frequency averages ${\bar{y}}_{k}$, the Allan deviation is commonly estimated using the standard non-overlapping estimator:

(3) $${\hat{\sigma }}_{y}\left(\tau \right)=\sqrt{\frac{1}{2(M-1)}\sum_{k=1}^{M-1}{\left({\bar{y}}_{k+1}-{\bar{y}}_{k}\right)}^{2}}.$$

ADEV is widely used because it compactly describes frequency stability as a function of averaging time τ and can be estimated directly from frequency measurements. However, its estimates become uncertain at long averaging times, can be affected by deterministic drift, and may not distinguish all noise types. For this reason, several Allan-like statistics are also used for specific analysis needs, including modified Allan variance (MVAR), Hadamard variance (HVAR), and parabolic variance (PVAR); their square roots—MDEV, HDEV, and PDEV, respectively—are reported analogously to ADEV [31].

Phase noise is a complementary frequency-domain measure of oscillator instability, typically expressed as the single-sideband phase-noise spectrum L(f) in dBc/Hz at a given offset from the carrier. In contrast to ADEV, which describes frequency stability in the time domain as a function of averaging time τ, phase noise describes the spectral distribution of phase fluctuations. Thus, phase noise is particularly useful for short-term phase/jitter analysis, whereas ADEV is more convenient for comparing frequency stability over specified averaging times [11,12,31]. Methods for migrating between time and frequency domains are also available, however, their use is limited due to the potential significant conversion inaccuracy [25,26].

### 2.3. Long-Term Stability Measures and Metrological Scope

For long observation times, oscillator stability is more commonly specified using deterministic or quasi-deterministic parameters rather than only ADEV. Aging, stress relaxation, contaminant adsorption, thermal memory, and electronics drift become important over such intervals [12,14,15]. In practical specifications, these effects are described by aging or frequency drift per day, month, or year, temperature stability over a specified temperature range, retrace after power cycling, and sensitivities to supply voltage, load, pressure, humidity, acceleration, vibration, and radiation. These measures are often more informative for long-term oscillator selection because they directly describe systematic environmental sensitivities and irreversible drift mechanisms, whereas ADEV may become dominated by deterministic trends and by the limited number of independent samples at very long averaging times.

Random frequency instabilities are described in IEEE Std 1139 [11], while measurement procedures and the determination of systematic instabilities due to environmental factors and aging, which are the subject of this article, are described in the IEEE Std 1193 guide [27]. Since this article focuses on the influence of environmental parameters on oscillator performance rather than on the metrology of phase noise and frequency stability, detailed measurement methods are not described here; they are discussed in detail by Rubiola [12].

### 2.4. Impact of Oscillator Instability on Sensor-System Accuracy

The effect of oscillator instability depends on the oscillator function in the sensor system. If y(t) is the fractional frequency deviation, the accumulated time error is, to first order,

(4) $$x\left(t\right)=\int_{0}^{t}y\left(u\right)\;du,$$

and the corresponding phase error at frequency $f_{0}$ is $\Delta \phi \left(t\right)=2\pi f_{0}x\left(t\right)$. Thus, a constant 1 ppm offset accumulates 86.4 ms of time error per day. In distributed sensing this produces timestamp and synchronization errors. In coherent detection and phasor-based measurement it produces phase rotation and loss of coherence [1,5,6].

For a sampled signal, a small clock error $\Delta t_{n}$ gives $\delta s_{n}≃\dot{s}\left(t_{n}\right)\Delta t_{n}$ [11], so the same timing uncertainty causes a larger amplitude error where the input changes rapidly. In frequency or period measurements referenced to an oscillator, the first-order relative measurement error has approximately the same magnitude as the reference fractional-frequency error, with the sign depending on the measured quantity and measurement convention. This is relevant to resonant sensors, SDR measurements, and Doppler-based sensing [8,9,10]. Consequently, oscillator selection must follow the sensor transfer function: accumulated offset and aging govern long-term synchronization, integrated jitter governs high-speed sampling, close-in phase noise governs coherent measurements, and reference-frequency accuracy governs frequency-encoded sensing.

## 3. Temperature Effects

**Figure 3:**
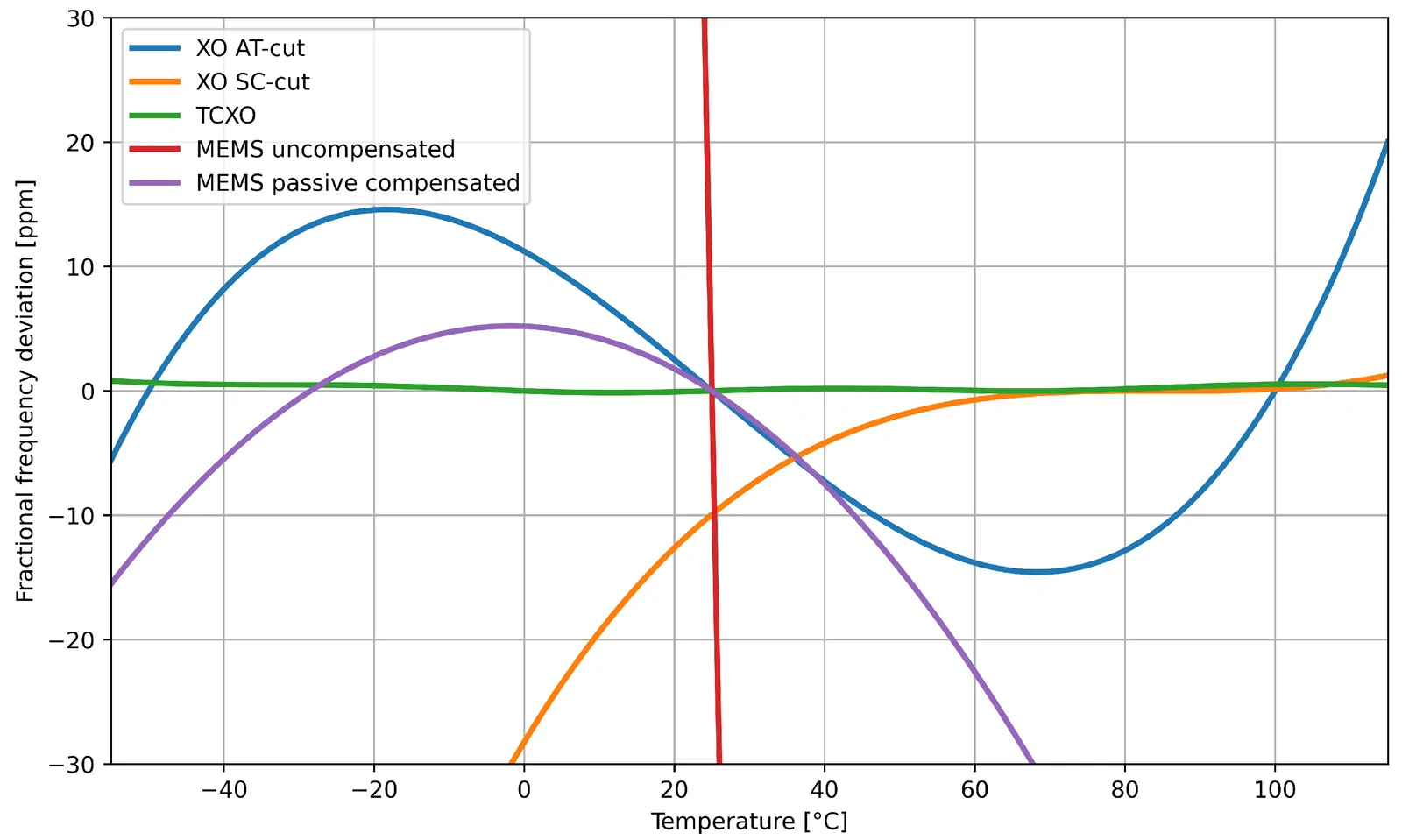
Illustrative simulated fractional frequency deviation of selected oscillator classes as a function of temperature [14,21,32,33].

Ambient temperature is one of the dominant factors affecting the performance of quartz and MEMS oscillators, because it simultaneously acts on the resonator, the mechanical parts (the package and the mounting of the quartz plate or silicon structure), and the electronics. Simulated characteristics of the individual oscillator classes, derived from data reported in the literature [14,21,32,33], are presented in Figure 3. OCXO and OCXO-class MEMS oscillators were omitted because of their different principle of operation.

### 3.1. Quartz Oscillators

In AT-cut quartz resonators, the frequency-temperature dependence has a shape close to a third-order curve, so it can be described by a polynomial expansion around a reference temperature:

(5) $$\frac{\Delta f}{f_{0}}=a_{1}\left(T-T_{0}\right)+a_{2}{\left(T-T_{0}\right)}^{2}+a_{3}{\left(T-T_{0}\right)}^{3},$$

where *T* is the current temperature, $T_{0}$ is the reference temperature, typically corresponding to the calibration point or the temperature at which the frequency equals $f_{0}$, and $a_{1}$, $a_{2}$, and $a_{3}$ are the linear, quadratic, and cubic temperature coefficients, respectively. This model describes the temperature sensitivity of a simple XO. The polynomial temperature characteristic of AT-cut quartz resonators, including the dependence of these coefficients on the cut angle and quartz type, has formed the basis of compensation design since 1956 [34]. Oscillator literature and manufacturer data sheets commonly report that uncompensated XOs exhibit frequency deviations on the order of tens of ppm over industrial temperature ranges, with the exact value depending on the resonator cut, load circuit, package, and operating conditions [15].

A TCXO reduces temperature error by incorporating compensation into the oscillator circuit. Temperature correction may be analog, digital, or mixed, but in typical implementations the idea is to change the load capacitance with a varactor or switched capacitors. This moves the operating point of the resonator to compensate for the influence of the temperature curve f(T). In this way, errors of more than ten ppm can be reduced to a single ppm, and in more complex designs, to a fraction of a ppm. In a high-frequency design, a closed compensation loop allowed a 100 MHz oscillator to achieve ±0.22 ppm over the −40–85 °C operating range [35]. When the analog correction scheme was extended with a multiplier generating higher-order terms, post-layout simulations predicted ±0.8 ppm over a wider temperature range of −40–95 °C [36]. An even wider operating range of −55–115 °C required time-domain accuracy-enhancement techniques, yielding ±1.4 ppm [37]. These studies show that improving one parameter, for example the operating temperature range, usually requires greater compensation complexity.

An OCXO also limits the influence of temperature on oscillator stability, but in a different way than a TCXO. Instead of correcting the full range of resonator temperature variations, it keeps the resonator in a thermally controlled chamber, typically in the 80–105 °C range [16], near a turnover point of the frequency-temperature curve of an SC-cut (stress-compensated) resonator (df/dT=0). Once the oven temperature stabilizes, the frequency error decreases from the ppm to the ppb level. In compact designs, thermal non-uniformity becomes the main limitation to stable operation. A heater integrated with the quartz oscillator allowed a miniature OCXO to achieve ±3 ppb over −40–85 °C [38]. Further miniaturization to a format intended for 5G synchronization (5 × 3.2 mm) operates over a wider ambient range of −40–95 °C, but with a lower stability of ±20 ppb [39]. In parallel, small OCXOs combining low short- and medium-term noise with low thermal sensitivity have been developed [40].

Another way to improve OCXO stability is segmented compensation, in which separate polynomials are fitted to selected temperature ranges. This approach reduced the error from ±4.29 ppb to ±0.153 ppb over −40–85 °C in a 20 mm × 20 mm design [41], while advanced miniature oscillators using fourth-order temperature control can achieve ppt-level stability [42]. These improvements, however, increase power consumption, warm-up time, and sensitivity to thermal gradients [43]. Adding an external thermoelectric cooler and thermoresistor, driven by a separate temperature-control module, to a complete OCXO shortened warm-up from about 3 min to less than 1 min while improving temperature stability [44].

### 3.2. Passive Temperature Compensation in MEMS Resonators

MEMS oscillators differ fundamentally from quartz devices. In silicon MEMS resonators, temperature affects the elastic moduli, stress, and geometry, making the temperature coefficient of frequency (TCF) a key design parameter (as shown in Figure 3 for uncompensated MEMS) [18]. Miniaturization improves shock resistance and integration but does not eliminate thermal sensitivity, because stresses are still transferred through the package, anchors, and material interfaces. Differences in coefficients of thermal expansion (CTEs), e.g., between silicon and glass, may generate thermomechanical stresses that degrade the quality factor, increase frequency drift, and, in extreme cases, cause delamination [45].

An early comprehensive overview of this technology was provided in a 2011 review comparing capacitive and piezoelectric transduction, oscillator noise sources, and the performance of MEMS devices relative to quartz resonators [17]. A more recent review of MEMS resonators describes progress in quality factor, motional impedance, temperature sensitivity, and initial frequency reproducibility [21]. The quality factor (*Q*) is one of the fundamental figures of merit for resonant systems and is defined as 2π times the stored energy divided by the energy dissipated per cycle. A higher *Q* generally enables lower phase noise and higher sensor resolution.

Temperature compensation in MEMS resonators is divided in this paper into passive and active methods, according to the classification proposed in Figure 1. Passive methods engineer the resonator itself, so that its material composition, doping, crystallographic orientation, geometry, or modal composition partially cancels the thermal drift. These methods require no temperature sensor, no control loop, and no additional power, and they act before any correction is applied. Active methods add sensing and feedback and consume power, but they reach substantially lower residual error.

#### 3.2.1. Composite Material Engineering

Silicon dioxide has a positive temperature coefficient of Young’s modulus, whereas that of silicon is negative. A composite structure combining the two can therefore be designed so that the thermal drifts of the constituents cancel each other. Depositing a thin silicon dioxide film on a single-crystal silicon resonator allowed the total frequency variation to be held below 200 ppm over −40–125 °C, giving a quadratic f(T) characteristic where the turnover temperature can be set by design [46]. Later characterization of the same device family put the linear temperature coefficient of the Young’s modulus of silicon dioxide at +179 ppm/°C and the residual quadratic TCF at about − 20 ppb/°C^2^ and proved the turnover temperature is tunable across −55–125 °C [47]. Monitoring over more than six months showed less than 2 ppm drift for most tested devices, although dielectric charging in the oxide emerged as a residual degradation mechanism. A further step was to distribute the oxide as an embedded matrix of pillars instead of a surface film, which cancels the linear TCF for both in-plane and out-of-plane modes. This reduces the total deviation to 83 ppm over −40–80 °C, 40 times lower than for the uncompensated silicon counterpart, while also improving the temperature stability of *Q* [48]. The pillar matrices showed no degradation of *Q* or insertion loss compared with bare silicon counterparts, and in the film-based composites *Q* increased by 15% to 50% depending on the design [47]. The lower thermal expansion coefficient and bulk modulus of the oxide reduce entropy generation through thermoelastic dissipation, which raises *Q* rather than lowering it.

#### 3.2.2. Degenerate Doping

Heavy *n*-type doping of silicon to a degenerate level, for example with phosphorus or arsenic, modifies the temperature coefficients of the elastic constants, thus affecting the temperature coefficient of frequency [18,49]. The effect is due to the redistribution of free carriers among the conduction-band minima under strain, which alters the elastic response of the crystal. Unlike composite compensation, this method needs no additional layer, and in its basic form it preserves a silicon-only structure. Measurements on *n*-type silicon wafers with carrier concentrations from $1.7\times 10^{19}$ to $7.5\times 10^{19}$ cm^−3^, doped with arsenic or phosphorus, showed that both doping level and temperature modify the elastic stiffness of silicon [50]. At a carrier concentration of approximately $2\times 10^{19}$ cm^−3^, the first-order temperature dependence of a shear-related elastic parameter becomes zero and increases to more than 40 ppm/K at higher doping levels. Consequently, the temperature coefficients of many extensional and flexural resonance modes can be tailored to provide first-order frequency compensation. These measurements were later summarized into design rules combining doping level, mode shape, and orientation to the achievable residual drift [51]. Analytical and finite-element models of *n*-doped silicon MEMS resonators predict turnover points near room temperature, with the first-order TCF reaching zero at doping concentrations of $0.5\times 10^{19}$ and $1\times 10^{19}$ cm^−3^ in the [100] length-extensional mode, the latter concentration being the one substantiated by the presented curves [49]. The same ability to set the TCF and the turnover temperature carries over to stacks that are not purely silicon. In thin-film piezoelectric-on-silicon resonators the turnover point remains a function of doping concentration and crystallographic orientation, while the thickness ratio of the silicon and the aluminum nitride film provides a further adjustment [52]. Doping therefore sets both the magnitude of the TCF and the temperature at which the turnover falls, with orientation and layer composition acting as fine-tuning parameters.

#### 3.2.3. Crystal Orientation

Orientation is the finer of the two fine-tuning parameters identified above, and throughout this section it denotes the in-plane alignment of the resonator with respect to the silicon crystal axes at a fixed wafer cut. Because the temperature coefficient of the individual elastic constants is different, the effective TCF of a vibration mode depends on how strongly the mode couples each of them, and therefore on the in-plane orientation of the resonator. With the doping level already fixed, rotating the device in the wafer plane moves the turnover point without changing the material. Doping, the choice of crystal orientation, and the selection of the vibration mode together limited the frequency deviation of bulk-mode capacitive resonators to about ±16 ppm over the range −40–85 °C [53]. Aligning length-extensional and square-extensional modes about 22.5° from the 〈110〉 direction in a degenerately doped (100) wafer gave ±20 ppm and ±16 ppm respectively. Combining the same alignment with heavy doping and an oxide composite layer in a scandium-doped aluminum nitride on silicon resonator gave ±21.5 ppm over the same range, with an unloaded *Q* of 10,017 [54] (corrected value in [55]).

#### 3.2.4. Geometry Engineering

Shaping the resonator and its supports in a way that competitive thermal stresses cancel each other out allows the same objective to be achieved from a mechanical point of view. In single-layer CMOS–MEMS plate resonators, folded anchors generate compressive or tensile thermal stress depending on the folding aspect ratio, and the two contributions have opposite signs. An optimized design used this to reduce the temperature coefficient by 100 times relative to straight anchor beams, placing the turnover point at 27 °C [56]. More recent modal-engineering approaches treat the TCF as a design parameter controlled by resonator geometry, redistributing the modal participation of the individual compliance constants. Applied to pseudo-Lamé resonators on ultra-highly doped silicon, that is, square plates carrying symmetry-preserving geometric modifications that retain Lamé-like behaviour while shifting the modal participation, this achieved a first-order coefficient of −0.39 ppm/°C, a second-order coefficient of −7.3 ppm/°C^2^, and a total frequency deviation of about 38 ppm over −20–70 °C, without additional fabrication complexity [57].

#### 3.2.5. Mechanical Coupling

Coupling two elements with different frequency–temperature characteristics makes the composite mode inherit a combination of their coefficients, which adds a design parameter absent from the four methods above. Coupling a square-extensional plate to a beam, or to a third plate vibrating in another bulk mode, allowed the TCF of the composite to be set by the volume and equivalent spring-constant ratio of the parts [58]. The minimum measured shift reached about ±10 ppm for an 18 MHz coupled square-extensional resonator over −40–85 °C. The method was then extended from the TCF to the turnover point itself. A tuning model expresses the turnover temperature of the coupled device as a weighted average of the turnover points of its constituents, in which each weight is the product of the effective mass and the second-order TCF of that constituent [59]. Coupling breathing-ring modes to length- or width-extensional modes in silicon doped with phosphorus to about $1.0\times 10^{20}$ cm^−3^ then moved the turnover point above the industrial temperature range, which is precisely the condition micro-oven-controlled oscillators require. Coupling can also raise the quality factor and lower the TCF at once. Two single-crystal silicon disks in a radial breathing mode, coupled to a piezoelectrically driven plate in a width-extensional mode, reached an unloaded *Q* of 80,695 at 25.202 MHz together with ±55 ppm over −40–85 °C [60]. Against a standalone resonator made under identical conditions, the averages show *Q* increased by 2.44 times, from 30,774 to 75,254, while drift is reduced by a factor of 2.5. Because the coupling ratio is defined lithographically, the mechanism composes with the doping, orientation, and composite techniques instead of competing with them.

### 3.3. Active Temperature Compensation in MEMS Oscillators

Active methods sense temperature, or a parameter that depends on it, and apply a correction based on that measurement. Three groups of compensation methods are recognized in this paper. In electronic compensation the correction acts on the output signal or on the oscillator loop, in micro-oven control the temperature of the resonator itself is regulated, and in nonlinear compensation the amplitude- or mode-dependence of the resonance frequency provides the correcting mechanism.

#### 3.3.1. Electronic Compensation

The simplest way is to add a temperature sensor and apply a stored correction, as in a quartz TCXO. Its accuracy is limited by the thermal coupling between the sensor and the active resonator region, which is what motivates using the resonator itself as the thermometer. In an example implementation, a calibration module built around a three-point interpolated discrete Fourier transform estimates the oscillation frequency without bias and feeds the correction back through a phase-locked loop, holding a 50 MHz MEMS oscillator within ±0.3 ppm over −40–85 °C, with the residual discrepancy between the measured and the actual frequency below ±0.4 Hz [61].

#### 3.3.2. Micro-Oven Control

Ppb-level stability in MEMS oscillators is most effectively achieved through active temperature compensation using local sensing and regulation. Integrated micro-ovens maintain the resonator near a constant temperature, while nearby sensors improve thermal tracking. A 2023 review summarizes such oven-controlled MEMS oscillator (OCMO) architectures, including their structures, sensors, and control systems [22]. Reported implementations achieved ±1.5 ppb while the ambient temperature was ramped from −40 °C to 60 °C over ten hours, with less than 30 mW of oven power. In a separate test at a constant ambient of 20 °C the same Lamé-mode device held an average change of about ±0.5 ppb over one week using 12 mW [20]. A dual-resonator platform reached ±100 ppb over −40–85 °C using a phosphorus-doped silicon resonator with a ScAlN layer and an adjacent temperature-sensing resonator on the same thermally isolated platform [62]. Increasing the operating range costs accuracy in the same way as in quartz TCXOs, and a dual-mode piezoelectric device phase-locked to the turnover point of its width-extensional mode gave ±190 ppb over the wider range −40–105 °C [63]. Heavy doping combined with a dual-mode distributed Lamé resonator allows the second mode to serve as an embedded thermometer, with heating and temperature-sensing resistors integrated into the resonator itself. Ovenized at its frequency turnover point of 250 °C, that device reached sub-ppb stability [64]. Ovenization also interacts with the package, and a wafer-level vacuum package (WLVP) with integrated micro-oven and thermal isolation reduced package stress to give a TCF of about 0.6 ppm/°C over −40–80 °C [65]. Implementing thermal regulation on the chip is not necessary. A two-stage scheme, in which an off-chip oven built from polyimide heating films under fuzzy PID control holds the oscillator at 35 °C to within ±1 m°C while the surrounding chamber is at −10 °C, achieved ±0.063 ppm [66].

The thermometer itself can also be improved. In dual-frequency resonance tracking (DFRT), two resonances of the same device are tracked digitally in a frequency-locked loop, and their ratio serves as a thermometer for regulating the resonator temperature. One vibration mode thus acts as a linear thermometer, which allows the resonator to be held at the turnover temperature of the main mode, approximately 65 °C for the Lamé mode. This reduced the gain of the sustaining electronics as a frequency-determining variable [67]. Macro-scale ovenization of a single-mode Lamé resonator on an external heating chuck reached a modified Allan deviation of 42 ppt at 85 s and sub-ppb performance up to 4800 s [68].

#### 3.3.3. Nonlinear Compensation

A third group of compensation methods avoids linear operation and uses the dependence of the resonance frequency of a strongly driven resonator on its vibration amplitude and on energy exchange with other modes. Amplitude–frequency compensation reduced drift to ±14 ppb during transient changes in ambient temperature [69]. A Duffing-based silicon oscillator, in which the resonance frequency depends on vibration amplitude because of mechanical nonlinearity, reached approximately ±1.88 ppb within a 1.47 °C range around the inflection point at 56 °C [70]. Compensating the second-order TCF as well as the first leaves a third-order temperature characteristic, whose insensitive region is around an inflection point, where $d^{2}f/dT^{2}=0$, rather than around a turnover point, where only df/dT=0. A related family uses internal resonance, in which two modes with a near-integer frequency ratio exchange energy and the driven mode saturates in frequency. The mechanism was first demonstrated as a way of stabilizing nonlinear micromechanical oscillators [71], and a 1:2 internal resonance in a stepped-beam resonator later improved frequency stability by more than 20 times [72]. Analysis of the underlying mechanism attributes the improvement to nonlinear frequency veering and to phase cleaning through modal synchronization [73].

Further improvement can be obtained through nonlinear mode coupling using blue-sideband excitation (BSE). The resonator is driven near the sum of two modal frequencies ($f_{i}$, $f_{j}$),

(6) $$f_{BSE}\approx f_{i}+f_{j},$$

which simultaneously excites coupled frequency components. If two resulting components exhibit nearly equal but opposite temperature coefficients, their combined output can largely cancel temperature-induced drift.

Using BSE, a stability of approximately 1.5 ppb at an averaging time of 1000 s was reported without power-intensive thermal control [74].

These figures were obtained under controlled laboratory conditions and should not be read as product-equivalent results, because each of these nonlinear schemes achieves its stability at the cost of a narrow operating window. They require the resonator to be held at a specific point in the amplitude–frequency plane, so performance depends directly on the drive level and on the gain and phase stability of the sustaining electronics, and the stabilizing effect disappears once the drive amplitude leaves the saturation region. Internal-resonance and BSE schemes additionally require a prescribed ratio between two modal frequencies, and the reachable stabilization range narrows as the intermodal mismatch grows [72]. Because modal frequencies are set by geometry and by the doping-dependent elastic constants, that mismatch is sensitive to process variation and generally has to be trimmed device by device. How narrow the window can become is visible in the reported results themselves, since the Duffing oscillator holds ±1.88 ppb within ±0.735 °C of its inflection point [70]. Driving a resonator this way also raises its dissipated power, so self-heating and mode coupling produce frequency shifts that may reinforce or partially cancel one another [75].

### 3.4. Product-Level Perspective on MEMS Timing Devices

Short-term stability, over averaging times from milliseconds to seconds, is determined primarily by the resonator quality factor and by the noise of the sustaining electronics. In the linear region, the classical feedback-oscillator noise model predicts that close-to-carrier phase noise improves as *Q* increases, because a steeper phase slope converts a given amount of amplifier noise into a smaller frequency deviation [12,76,77,78]. The reachable quality factor is not in itself what separates the two technologies. The phonon-interaction limit on the f×Q product below 1 GHz is predicted to be between $1.8\times 10^{13}$ and $3.2\times 10^{13}$ Hz for quartz, between $2.5\times 10^{13}$ and $3.9\times 10^{13}$ Hz for silicon, and between $0.9\times 10^{13}$ and $2.5\times 10^{13}$ Hz for aluminum nitride, so the maxima obtainable with MEMS are of the same order as those of quartz [17]. What differs in practice is how closely a given design approaches that limit, and that is set by anchor and tether losses and by the package.

Enhancement of *Q* is a route to short-term stability, and much recent MEMS work targets those parameters. Mechanical optimization has achieved good results: a silicon BAW resonator with anchors aligned with nodal locations reached $Q=1.8\times 10^{6}$ at 1.25 MHz and an Allan deviation of 36 ppt at 400 ms, although without active thermal compensation ambient fluctuations degraded that to 0.3 ppm at two hours [79]. Phononic-crystal structures suppress anchor loss while remaining compatible with ovenization; a honeycomb-lattice micro-oven implementation raised the maximum unloaded *Q* to 42,784 and reached 0.62 ppb at an averaging time of 1.02 s under micro-oven control [80]. Measurements on five oscillators referenced to different modes of a single AlN-on-Si resonator show that this scaling is not absolute: the highest-*Q* mode (Q=18,600) gave the lowest close-to-carrier phase noise and an Allan deviation of $5.5\times 10^{-9}$ at τ=1 s, but the best short-term stability, $4\times 10^{-9}$, came from the Q=4230 mode [81]. Once the resonator is driven into the nonlinear region the linear prediction no longer holds, and the flicker- and random-walk-frequency noise levels depend on the nonlinear operating point instead of on *Q* alone [82].

Temperature stability over the specified operating range is determined primarily by the combination of passive and active TCF compensation. The practical division of tasks in current devices is that passive engineering reduces the residual deviation to the order of 10 ppm, after which an electronic or micro-oven loop is required to reach the ppb level.

Long-term stability and aging depend strongly on the resonator environment, and therefore on the encapsulation. Because the surface-to-volume ratio of a MEMS resonator is large, small changes in surface contamination shift the frequency measurably, and badly packaged devices drift significantly more than well-sealed ones. The solution to this problem is thin-film wafer-level vacuum encapsulation by sealing the resonator in a clean, high-vacuum cavity at high temperature, before any back-end processing. Encapsulated resonators showed no measurable change in cavity pressure over more than 10,000 h and after 600 thermal cycles [83]. In a separate frequency-stability study, no trend of resonant frequency shift could be detected during 10,000 h of operation and 680 temperature cycles between −50 and 80 °C, within a measurement uncertainty of a few ppm [84]. The epitaxially sealed variant of the process, known as epi-seal, has been reported as implemented in commercial MEMS timing technology [85]. Both the ovenized resonators cited above [20] and the ppt-level holdover reference [67] were built in it, confirming that packaging is a necessary factor for long-term performance. Encapsulation does not remove the influence of mechanical design. Among wafer-level vacuum-encapsulated single-crystal silicon devices, BAW resonators drifted by less than 0.1 ppm/month, whereas flexural resonators made in the same process drifted from 4 to more than 500 ppm/month depending on the anchoring, an effect attributed to package-related stress [86].

Commercial MEMS oscillators reflect these parameters and have advanced beyond simple XO replacements. According to manufacturer data sheets, the SiT5811 provides ±1 ppb stability over −40–95 °C, a typical frequency slope of ±0.01 ppb/°C, and aging of ±0.1 ppb/day, specified inclusive of an 8 °C temperature change following the ITU-T profile with 1 m/s airflow [87]. In dynamic applications, mechanical robustness may be as important as nominal stability: the SiT7101 specifies a typical acceleration sensitivity of 0.01 ppb/g and 20,000 g shock resistance [88], while the SiT5543 offers 0.01 ppb/g sensitivity and ±5 ppb stability [89]. These product specifications are useful for selection but are not independent experimental validation.

The quantitative results discussed in this section are collected in Table 2. The reported metric, the temperature range or averaging time to which it applies, the thermal control and power required, and the level at which the result was evaluated are listed in separate columns. A first-order temperature coefficient, a bound on the deviation from nominal over a stated range, and an Allan deviation at a stated averaging time quantify different properties, and they belong to two distinct measurement frameworks. Allan deviation describes the random frequency instabilities defined in IEEE Std 1139 [11], whereas temperature coefficients and deviations over a stated range describe the systematic instabilities whose measurement procedures are given in IEEE Std 1193 [27]. A value measured on a bare resonator in a laboratory setup is also not equivalent to a specification guaranteed for a packaged product.

### 3.5. Temperature Gradients, Thermal Lag, and Self-Heating

Time-dependent thermal effects are often grouped together as temperature sensitivity, but four mechanisms with different signatures should be separated, because each calls for a different remedy. Temperature gradient means that different regions of the resonator are at different temperatures at the same time, so the frequency responds to a distribution instead of a single value. Thermal lag, by comparison, is a purely dynamic error. The temperature sensor and the active resonator region have different thermal time constants, so under a varying ambient the compensation circuit corrects for a temperature the resonator has not yet reached, which makes the error vanish in steady state and grow with the rate of temperature change. Thermal hysteresis is a history effect instead. The frequency at a given temperature differs between ascending and descending ramps because the mechanical, material, package, and circuit state depends on the path taken to reach that temperature, so no correction based on the instantaneous temperature can remove it. Self-heating differs from all three in being internal rather than external, since the resonator dissipates part of the drive power and operates above ambient by an amount set by the drive level and by the thermal resistance to the package.

The static f(T) characteristic is insufficient under time-varying ambient temperature. Because the sensor, package, board, and resonator have different thermal time constants, the compensation circuit may respond to the sensor temperature rather than that of the active resonator region. The resonator should therefore be treated as a structure with a nonuniform temperature distribution. A model of this thermal lag is given by [93]:

(7) $$\frac{d}{dt}x\left(t\right)=A\;x\left(t\right)+b\;T_{e}\left(t\right)$$

where x(t) is an *N*-element vector containing the time-dependent temperatures $x_{i}\left(t\right)$ of the discretized resonator elements, and $T_{e}\left(t\right)$ is the instantaneous ambient temperature surrounding the resonator. The N×N matrix A describes heat exchange and thermal coupling between the individual resonator elements, whereas the *N*-element vector b represents heat transfer between the resonator and its environment. The elements of A and b depend on the material properties, heat-transfer parameters, resonator geometry, and the adopted spatial discretization method. This distributed-temperature model is particularly important when local heat sources produce spatial temperature gradients across the resonator. For quartz crystal microbalances (QCMs), such gradients affect the resonance frequency strongly enough that they have to be compensated as well as the mean temperature [43]. Compensation based on both the mean temperature and the temperature gradient reduced the residual error to below 5% of the original thermal disturbance [94].

Although QCM devices function primarily as sensors rather than frequency references, the same mechanism matters in precision oscillators, where it appears as nonuniform oven temperature distribution, cooling of the package by airflow, and controller lag. For this reason the latest small OCXOs are tested under the realistic, dynamic temperature profiles in which they are ultimately expected to operate, rather than in a static climate chamber alone. For instance, the ROD2522S2 design achieved a time error below 1.5 μs over 24 h of holdover under a 40 °C cycle with a 1 °C/min gradient, following a 52 h GNSS-disciplined aging-learning phase, with a frequency hysteresis smaller than $\pm 5\times 10^{-11}$ at the same gradient [95]. Modeling the transient thermal response of the resonator together with the oven control loop shows that maintaining ADEV on the order of $10^{-13}$ at 100 s requires restricting ambient temperature instability to below 10 mK [96], and active stabilization of an environmental chamber to ±25 mK, ±0.1% relative humidity, and ±12 Pa improved the stability of ultrastable quartz oscillators by a factor of about 100, to $2\times 10^{-13}$ at 1–10^5^ s [97].

Temperature gradients also affect MEMS oscillators. A wafer-level vacuum package with an integrated micro-oven and thermal isolation reduced package stress and achieved a TCF of about 0.6 ppm/°C over −40–80 °C [65]. Adaptive mode-coupling stabilization, in which a nonlinear low-frequency resonator regulates a linear high-frequency one through phase control, improved frequency stability nearly 700 times at an averaging time of 1000 s, and by over 1000 times at 802 s under varying ambient temperature [91]. In a curved piezoelectric MEMS resonator tested from 25 °C to 75 °C, using the frequency difference between two vibration modes yielded an Allan deviation of 127 ppb at 1.45 s, while operating a further mode at its temperature-insensitive extremum near 55 °C gave 2.0 ppb at 2.51 s [90]. Thus, practical performance depends on the thermal robustness of the entire module, not only on the static f(T) characteristic.

Self-heating requires separate treatment, because it couples the electrical operating point of the oscillator to its thermal one. Part of the supplied power is dissipated within the driven structure, which raises its mean temperature above ambient in proportion to the drive power and to the thermal resistance between the resonant body and the package. The resulting frequency offset therefore follows the drive level rather than the environment, appearing as a start-up transient and as a drive-level-dependent error. In aluminum nitride contour-mode resonators, this thermal nonlinearity distorts the frequency response at elevated drive levels [98]. It matters most in dual-mode devices, where one mode is driven while the other serves for sensing or as a reference. Under dynamic piezoelectric excitation the signal in one mode shifts the frequency of the other through the combined action of self-heating and mode coupling, and the two contributions may act in opposite directions [75]. Holding the resonator at a carefully selected TCF point with an on-chip micro-oven then allows the mode-coupling shift to cancel the self-heating shift, which turns a parasitic interaction into a stabilization mechanism.

A summary of the effects of thermal dynamics on oscillator stability is given in Table 3.

### 3.6. Thermal Hysteresis

**Figure 4:**
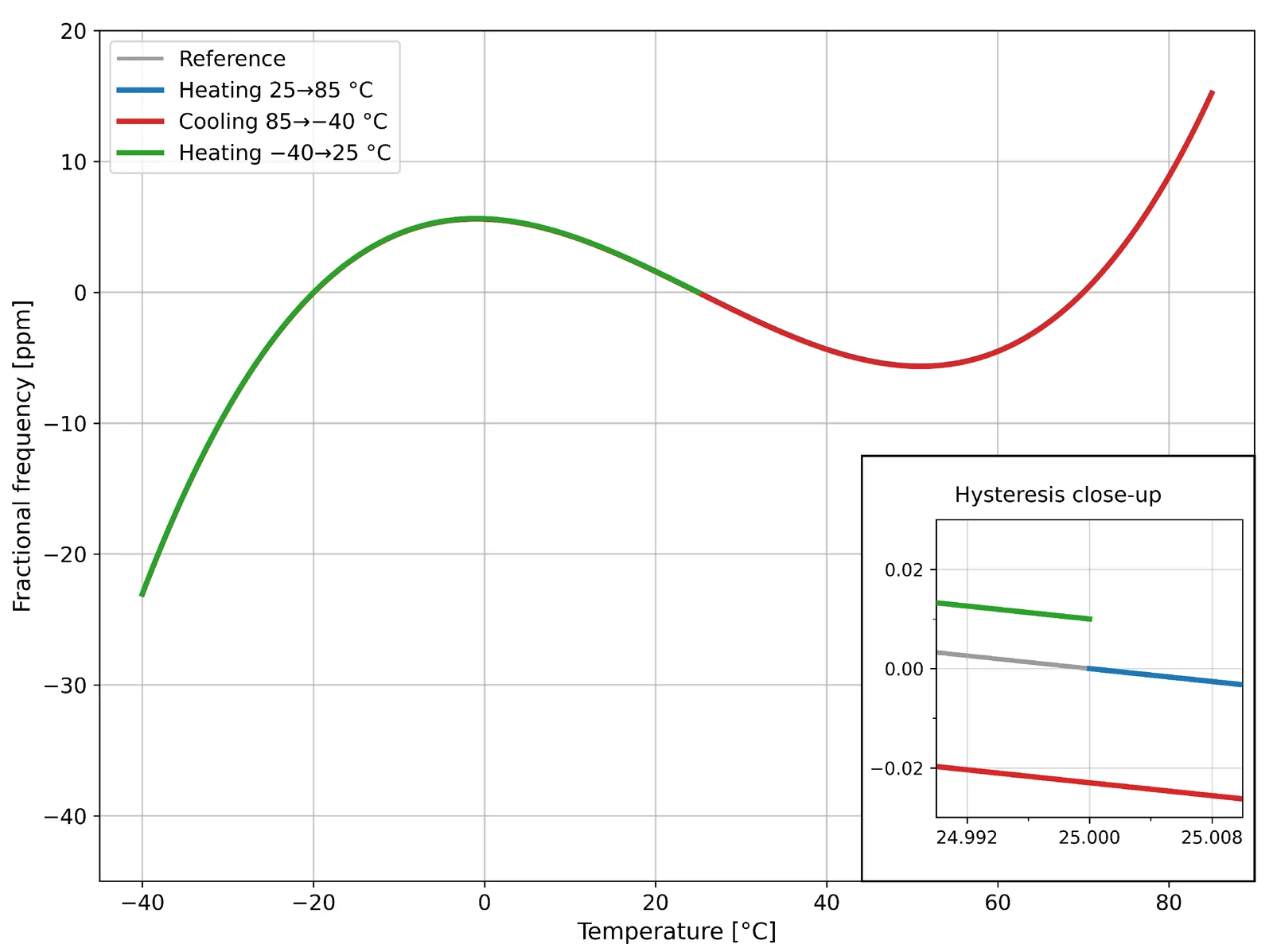
Illustrative simulated thermal hysteresis of the fractional frequency–temperature characteristic in AT-cut resonators.

Compensation based only on the current temperature assumes a unique frequency for each temperature. In practice, oscillator frequency also depends on environmental history, as illustrated in Figure 4 [13]. After heating and cooling, residual stresses in the resonator and mechanical structure may cause different frequencies at the same temperature during ascending and descending ramps. Thermal hysteresis in quartz resonators may result from stress changes, impurity migration, lattice defects, circuit hysteresis, and thermal gradients [23]. A related memory effect is frequency retrace, defined as the incomplete return of frequency after power-off, cooling, and reheating to the nominal operating temperature. In high-stability OCXOs, retrace is specified after power cycling. In a dual-mode digital OCXO, the frequency deviation after a one-day off-period was about 2 ppb, and it returned to within ±0.2 ppb of the pre-shutdown value after about 10 h [101]. Measurement procedures for retrace, warm-up time, and environmental sensitivities are described in IEEE Std 1193 [27]. Their interpretation requires care, because the sensor or package temperature may differ from that of the active resonator region [99]. Such effects often become evident only after static compensation errors have been reduced.

Further improvement of TCXOs requires transition from the static f(T) model to f(T,H), where *H* represents thermal history. In cyclic tests, trajectory-aware compensation reduced hysteresis error from about 700 to 150 ppb over −40–80 °C [32]. This idea has been extended using machine learning: artificial neural networks have modeled the dependence of frequency on the preceding temperature trajectory [100], while a long short-term memory (LSTM) network achieved an RMS error below 0.05 ppm for combined temperature and hysteresis effects [102]. Such memory-based compensation becomes practical once the static temperature error is sufficiently reduced for hysteresis to become significant.

Thermal hysteresis also occurs in MEMS resonators and depends strongly on their mechanical architecture. Hermetically sealed resonators with a single central anchor showed no measurable hysteresis after 460 cycles over −50–80 °C [103], and the same conclusion was reached over 680 cycles in wafer-scale film-encapsulated devices [84]. In contrast, a multi-anchor piezoresistive MEMS oscillator tested over 40–300 K exhibited hysteresis in both resonance frequency and quality factor, and that hysteresis grew as the support beams were shortened from 100 to 30 μm [104]. For the shortest beams the effect on the quality factor was severe: at 220 K the measured *Q* was more than twenty times larger on the descending ramp than on the ascending one, so the same nominal temperature corresponded to two very different linewidths. Repeated cycling reduced both effects, almost entirely for the frequency, which points to stress relaxation in the anchors.

Retrace is a specified parameter for quartz OCXOs, and in well-encapsulated silicon devices it is relatively small. Repeated power and thermal cycling of epi-seal resonators produced no observable change in resonant frequency [105]. Memory effects in MEMS therefore originate mainly in the package and the anchoring, whereas in quartz contamination redistribution and changes within the crystal itself contribute directly. MEMS hysteresis is thus more complex than in quartz devices, but it can be mitigated through appropriate mechanical design and encapsulation.

A summary of the effects of thermal hysteresis and retrace is given in Table 4.

In summary, temperature-induced frequency variation remains one of the dominant environmental limitations in both quartz and MEMS oscillators, although its physical origin differs between the two technologies. In quartz devices, it is governed primarily by the intrinsic frequency-temperature characteristic of the crystal and by thermomechanical effects associated with mounting and packaging, whereas in MEMS resonators the temperature dependence of elastic properties, structural stress, geometry, anchors, and material interfaces all contribute to the resulting frequency shift. Passive compensation can substantially reduce the intrinsic temperature coefficient without additional operating power, but the highest stability requires active electronic correction or thermal regulation. These approaches can reduce temperature-induced errors from the ppm range to the ppb level or below, at the cost of increased calibration and circuit complexity, power consumption, warm-up time, or sensitivity to process variations and the selected operating point. At this level of performance, further improvement is increasingly limited not by the static f(T) characteristic itself, but by temperature gradients, thermal lag, self-heating, hysteresis, and package-related stresses. The principal remaining challenge is therefore to maintain low frequency sensitivity under dynamic temperature conditions while minimizing power consumption and calibration effort and preserving long-term stability at the packaged oscillator level.

## 4. Aging and Long-Term Drift

Aging is a slow, partly irreversible frequency drift that occurs even under nominally constant temperature, supply, and load. It results from combined processes in the resonator, mounting, package atmosphere, and oscillator electronics. In quartz devices, the main mechanisms include adsorption and desorption of surface molecules, contaminant redistribution, electrode-mass and stress changes, relaxation of manufacturing and mounting stresses, and slow changes in the piezoelectric material [15,28]. Package leakage, moisture, deformation and stress relaxation of bonding materials, as well as drift of passive components, active-device bias, and drive level, introduce additional errors. Oscillator aging is therefore more complex than resonator aging alone [14]. High temperature accelerates many of these processes and is used in accelerated aging tests. In OCXOs, stable thermal conditions can make the resulting drift more regular and easier to model.

Practical aging compensation increasingly relies on monitoring each oscillator rather than using a universal coefficient. Since individual characterization is impractical in mass production, a statistical model applied to a batch of OCXOs improved aging performance by one to two orders of magnitude [106]. An even more hardware-oriented approach measures the phase shift between the resonator pins, as a change in the resonance parameters manifests directly in the loop phase. This method makes it possible to track OCXO drift without an external reference, improving long-term stability from the resonator’s own signal [107]. In satellite systems, combined temperature-aging models and clock-drift filtering allowed a low-cost ±3 ppm TCXO to replace a ±0.01 ppm OCXO while maintaining timing error below 0.8 ms per day [108].

In MEMS oscillators, aging arises from mechanisms different from those in quartz resonators. Their small mass and high surface-to-volume ratio increase sensitivity to adsorption, package hermeticity, and residual gas in the capsule [17,18]. At high temperatures, electrode degradation, including titanium nitride oxidation and aluminum migration, can permanently alter mass, stress, and effective stiffness, leading to long-term frequency drift. Changes in package pressure or gas composition may also affect damping, *Q*, and heat transfer. Integration with an IC enables parameter monitoring and digital compensation. Commercial devices report aging of ±0.1 ppb/day for the SiT5811 and SiT5812, specified inclusive of an 8 °C temperature change following the ITU-T profile with 1 m/s airflow [87,92], and ±150 ppb over 20 years for the low-power SiT5543 [89].

Among the studies reviewed here, directly comparable aging data for the two technologies are rare, because the two technologies are seldom evaluated under a common protocol. In a side-by-side characterisation of commercial quartz and MEMS oscillators aged for 2928 h in a common test system, the quartz devices drifted by 0.02 to 0.25 ppm, consistently downward, whereas the MEMS devices drifted by 0.19 to 2.83 ppm [109].

Recent studies indicate that the long-term holdover stability of hermetically sealed MEMS oscillators can rival even that of chip-scale atomic clocks (CSACs) under laboratory conditions. Combining ratiometric stabilization with an amplitude-based frequency-locked-loop (FLL) was reported to allow a 268 MHz MEMS clock to achieve MDEV of about $8\times 10^{-12}$ at an averaging time of 8 h, with a possible drift of −4.1±2.5 ppt/day [67].

Table 5 summarizes aging mechanisms and compensation methods.

Overall, aging and long-term drift arise from slowly evolving physical and chemical changes in the resonator, mounting, package, and sustaining electronics, making them inherently more difficult to compensate than reversible environmental effects. In quartz oscillators, contamination, mass redistribution, stress relaxation, and package- and electronics-related changes are the dominant mechanisms, whereas MEMS devices are additionally influenced by their high surface-to-volume ratio, surface adsorption, package atmosphere, and long-term changes in structural and electrode materials. Aging can be reduced through improved materials, processing, hermetic packaging and stabilization procedures, while oscillator-specific modeling, parameter monitoring, and digital correction can further improve long-term stability. In compensated devices, these measures can reduce aging rates to below 0.1 ppb/day [87] under standardized thermal-cycling and airflow test conditions, at the cost of additional monitoring electronics, calibration effort, or continuous oven power. For quartz oscillators, the relevant mechanisms and mitigation procedures are comparatively well established, whereas long-duration MEMS data obtained under standardized and directly comparable conditions remain more limited. The principal remaining challenge is therefore not only to minimize aging itself, but also to predict device-specific long-term drift reliably and to distinguish intrinsic aging from slow changes caused by temperature, package conditions, electronics, and other environmental factors.

## 5. Acceleration, Vibration, and Shock

Every resonator has mass and elastic mounts, so acceleration changes the stresses in the vibrating structure. For small disturbances, this relationship can be described by the dot product of the acceleration-sensitivity vector Γ and the acceleration vector, which links acceleration to the fractional frequency change [27,111]:

(8) $$\frac{\Delta f}{f_{0}}=\Gamma \cdot a_{g},$$

where $a_{g}$ is the acceleration expressed in units of standard gravity, so that the components of Γ are given in ppb/g. The vector Γ defines the magnitude and direction of the oscillator’s acceleration sensitivity, so the resulting frequency shift depends on device orientation. Constant acceleration produces a static offset, whereas vibration causes time-varying frequency modulation that degrades spectral purity and short-term stability. Sensitivity along a given axis can be measured using the 2-g tipover test, in which the oscillator is rotated by 180° in the gravitational field. This first-order model is adequate for small loads, but shock conditions may require second-order nonlinear analysis [112]. Acoustic noise produces similar effects by exciting the package and printed circuit board (PCB) through pressure fluctuations. Therefore, acceleration, vibration, shock, and acoustic sensitivity are commonly considered together in oscillator environmental testing [15,27].

Static loads transmitted through mounts, solder joints, or PCB flexure can also shift the resonator frequency. In AT-cut resonators, the force–frequency effect depends on the force application point and resonator orientation [113]. Consequently, oscillators with good laboratory stability may perform substantially worse under dynamic conditions, as observed in flight tests [4].

For miniature AT-cut quartz resonators, the acceleration sensitivity is typically on the order of 2 ppb/g. In applications requiring low phase noise under vibration, values below 1 ppb/g, or even approaching 0.1 ppb/g, may be required. The acceleration sensitivity of an oscillator can be reduced by pairing two crystals with oppositely directed sensitivity vectors so that their individual mechanical responses partially cancel. In oscillators spanning the 10–40 MHz frequency range, this method achieved results below 0.1 ppb/g [111]. The same goal can be reached using geometry. Shifting the electrode region relative to the anchor in a 25 MHz oscillator design reduced the sensitivity to approximately 0.5 ppb/g [114]. A resonator operating on the third overtone, tested under vibration excitations in the 200–2000 Hz range, achieved an acceleration sensitivity of about 0.3 ppb/g [115]. Active compensation can also use an integrated accelerometer to digitally correct OCXO frequency under conditions ranging from satellite microvibrations to aircraft-carrier launch [116].

MEMS oscillators benefit from their low moving mass. In a Lamé-mode resonator, acceleration sensitivities of 2.1 ppb/g in-plane and 3.8 ppb/g out-of-plane were measured. A central anchor reduced the latter to 0.74 ppb/g. Drop tests revealed a maximum transient frequency deviation of −7.7 ppm measured 1.7 ms after an 11,000 g impact. Notably, the oscillator continued to operate nominally even after experiencing eight shocks at 5000 g and four at 11,000 g [117].

Commercial MEMS oscillators can outperform many quartz devices in specified mechanical robustness. Manufacturer data for the SiT7101 give a typical acceleration sensitivity of 0.01 ppb/g and a 20,000 g shock rating, while the SiT5543 has an option specified at 0.01 ppb/g [88,89]. These are product specifications, not independent measurements, and low acceleration sensitivity does not by itself imply low phase noise under vibration. Long-term reliability still depends on anchors, package, mounting, and the applied shock waveform [45].

A summary of the effects of acceleration, vibration, and shock is given in Table 6.

Acceleration, vibration and shock affect oscillator stability primarily through mechanically induced stress in the resonator, its supports, package and PCB. The resulting frequency shift depends strongly on the geometry and orientation of the device. In quartz oscillators, acceleration sensitivity can be substantially reduced through resonator and electrode design, mechanical cancellation techniques, or active feedforward correction. MEMS oscillators, on the other hand, benefit from their small moving mass and can achieve high resistance to severe shock through optimized anchoring and protective packaging. These measures can reduce acceleration sensitivity from a typical baseline near 2 ppb/g [15,27,118] to as low as 0.01 ppb/g in ruggedized products [88,89]. However, these improvements may require more complex resonator structures, additional sensing and correction electronics, or specialized packaging. Furthermore, low acceleration sensitivity alone does not guarantee low phase noise under vibration. Therefore, the principal remaining challenge is to simultaneously achieve low acceleration sensitivity, low vibration-induced phase modulation and high shock robustness without compromising thermal performance, spectral purity, power consumption or long-term mechanical reliability.

## 6. Pressure and Humidity

A hermetic resonator enclosure significantly limits the influence of moisture and gases, but it does not completely isolate the oscillator from atmospheric effects. Moisture can alter leakage currents and parasitic capacitances on the printed circuit board, while the gas inside the package affects damping, heat transfer, and warm-up time. In OCXOs, operation in nitrogen, helium, and vacuum has been studied in relation to energy consumption, as the gaseous medium modifies heat transfer between the heater, resonator, and package [16].

In QCM sensors, the influence of moisture is exploited intentionally. Reported designs achieved sensitivities of 116.2 Hz and 82.40 Hz per percentage point of relative humidity using a humidity-sensitive capacitor and a graphene oxide layer, respectively [119,120]. These figures illustrate the scale of quartz surface sensitivity, but they cannot be transferred directly to oscillators enclosed in hermetic packages.

For vacuum-packaged MEMS oscillators, helium used during testing poses an additional risk because it can permeate through silicon and wafer-bonding interfaces. This degrades the internal vacuum, reduces the quality factor and loop gain, and may disturb oscillator operation [121,122]. Slow gas leakage can be modeled as an exponential rise in the reciprocal of the quality factor over time, allowing the time required to reach a stable cavity condition to be estimated [110].

In subsea and extreme high-pressure applications, heavy steel or titanium capsules are often replaced by pressure-compensating media such as resin, gel, or oil. These materials prevent short circuits and equalize pressure, but they do not remove the mechanical action on the components themselves [123]. As a result, conventional metal or ceramic oscillator packages can deform, changing stray capacitances and transferring stress to the resonator [15]. Under repeated pressure cycling, this direct exposure produces increased frequency and amplitude variability, causing long-term drift or even the complete failure of MEMS oscillators [124].

The reported test methodology was built around a test board placed in an oil-compensated housing and a chamber rated up to 600 bar [123]. Quartz oscillators operating at 8, 16, 32, and 50 MHz showed frequency-dependent pressure sensitivity: lower-frequency units remained relatively stable up to about 400 bar, whereas the 32 MHz and 50 MHz oscillators became unstable above approximately 200 bar and 20 bar, respectively. All devices failed at 425 bar, with package buckling observed after testing. This demonstrates that hydrostatic pressure in an oil-compensated system directly affects the oscillator despite its native factory packaging.

A comparison of quartz and MEMS oscillators showed that their pressure responses differed in both magnitude and direction. The 8 MHz and 24 MHz MEMS devices exhibited decreasing frequency with increasing pressure and residual shifts after repeated cycles, while the 50 MHz MEMS oscillator degraded after the second maximum-pressure cycle, suggesting possible decompression-induced damage [124]. Quartz oscillators showed much smaller changes. Although no explicit pressure-sensitivity coefficients were reported, the frequency maps indicate a negative response of tenths of a ppb per hPa for the tested MEMS devices, approximately one order of magnitude stronger than for quartz. These effects reflect the susceptibility of the entire oscillator assembly, including the resonator, substrate, package deformation, and other components.

The pressure sensitivity of oscillators is significantly less thoroughly described than their response to other stability-determining parameters. Earlier studies reported that the medium- and long-term stability of selected quartz oscillators can depend on atmospheric humidity and probably also on pressure. The suggested mechanisms included changes in thermal gradients, dielectric properties, and leakage between circuit elements [125]. More recent work typically concerns specialized conditions [123]. Studies of miniature hermetically sealed OCXOs further show that packaging, sealing, and thermal design are integral to frequency stabilization, although they do not directly quantify sensitivity to normal atmospheric-pressure variations [126]. Stable pressure conditions have also been identified as important for high oscillator stability [97]. Similar residual sensitivity occurs in vacuum-packaged MEMS resonators, where mismatched temperature and pressure coefficients between coupled resonators can limit frequency stability [127].

A summary of the effects of pressure and humidity is given in Table 7.

Pressure and humidity act mainly through package stress, cavity damping, heat transfer, leakage, and dielectric paths [125,127]. Hermetic sealing limited these effects under the reported conditions but did not guarantee immunity to helium permeation [121,122] or extreme hydrostatic load [123,124]. Given this sparse, sample-specific evidence, neither a representative achievable-improvement figure nor a generalizable implementation penalty can currently be stated with confidence—this scarcity of standardized, comparable data is itself the principal remaining challenge, so pressure coefficients should be measured per package and mounting rather than assigned generically to quartz or MEMS.

## 7. Power Supply, Load, and Loop Electronics

The resonator is a passive element and requires sustaining electronics in a closed feedback loop to form an oscillator. Therefore, the output frequency depends not only on the quartz plate but also on the sustaining circuit, load and parasitic capacitances, PCB layout, and receiver impedance [13]. These elements modify the reactance seen by the resonator and shift the oscillation frequency by altering its electrical equivalent model [24]. Recent XO models likewise show that the resonator temperature characteristic alone is insufficient, because the RLC parameters and circuit capacitances also affect oscillation [128].

The load capacitance $C_{L}$ is of particular importance, because its controlled variation can be used as a temperature-compensation mechanism. One example is low-power 32.768 kHz TCXOs that implement compensation by switching the load capacitance with ΔΣ modulation, achieving an accuracy of ±4.2 ppm over the −20–85 °C range at a power consumption of 43 nW at room temperature [129]. The load capacitance is thus simultaneously a parameter that enables correction and a potential error source.

**Figure 5:**
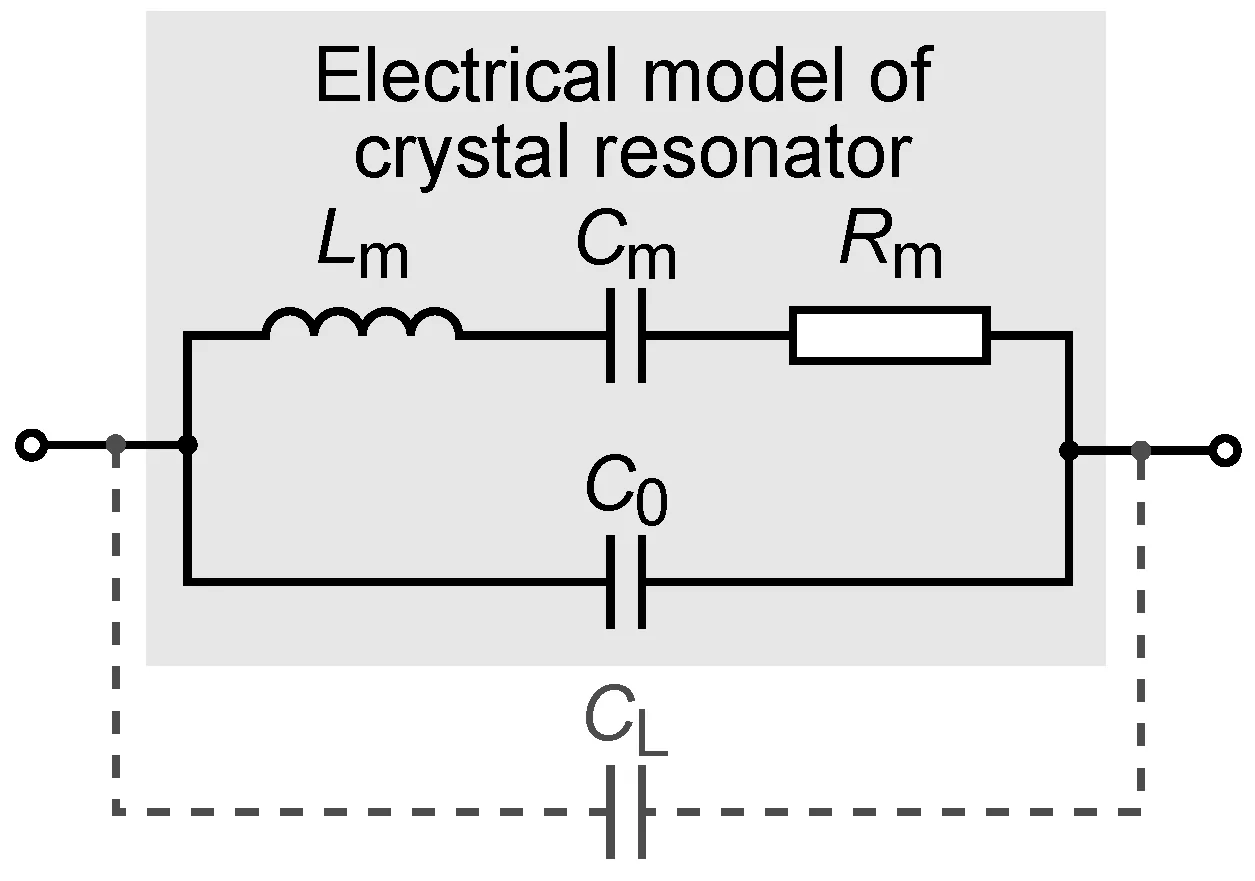
Equivalent electrical model of a quartz crystal resonator with load capacitance $C_{L}$.

Figure 5 shows the equivalent electrical model of a quartz resonator, consisting of the motional branch $R_{m}$, $L_{m}$, and $C_{m}$, connected in parallel with the shunt capacitance $C_{0}$. The motional branch determines the series resonant frequency $f_{q}$. Owing to $C_{0}$, the unloaded parallel resonant frequency $f_{p}$ is slightly higher than the series resonant frequency $f_{q}$. Their relative separation can be approximated as [35]:

(9) $$\frac{f_{p}-f_{q}}{f_{q}}\approx \frac{C_{m}}{2C_{0}}.$$

When the resonator operates with an external load capacitance $C_{L}$, the relative shift of the resulting loaded resonance frequency $f_{L}$ is given by

(10) $$\frac{f_{L}-f_{q}}{f_{q}}\approx \frac{C_{m}}{2(C_{0}+C_{L})}.$$

Thus, $f_{q}<f_{L}<f_{p}$, while the nominal oscillator frequency $f_{0}$ is typically close to $f_{L}$ at the specified load capacitance $C_{L}$. The load-pulling range depends on the ratio of $C_{m}$ to $C_{0}+C_{L}$. Increasing $C_{L}$ reduces sensitivity to parasitic capacitance variations, whereas decreasing it increases frequency pulling. Consequently, uncontrolled load variations may shift the frequency of an XO [24], while in a TCXO the same effect is deliberately used for temperature compensation through varactors or switched-capacitor networks [37,129,130].

In MEMS oscillators, load capacitance has a different role than in quartz devices. The capacitance connected to the oscillator output mainly affects the buffer, increasing rise time, switching current, and possibly output jitter, but usually does not pull the nominal frequency. More important are the capacitances inside the resonator path and the oscillation loop. In a capacitive MEMS oscillator, the mechanical motion of the structure is converted into a very small electrical signal. Consequently, the electrode, parasitic, amplifier input, and shunt capacitances affect the oscillator performance. In a Pierce circuit with a MEMS resonator, the external capacitances indeed influence the oscillator stability and the power consumption of the circuit. The feedthrough capacitance (the capacitance between the output and the input) has the largest impact on the operating characteristics of the oscillators, compared with the capacitances to ground. This translates into higher power consumption and greater sensitivity of stability to the phase and noise of the electronics. In MEMS, therefore, reducing the feedthrough and parasitic capacitances is not merely a matter of load matching, but one of the conditions for achieving low power and good frequency stability [131].

Another approach to reducing sensitivity to phase errors in the electronics caused, e.g., by temperature variations or aging of the circuits, is to replace a conventional phase-locked loop (PLL) with a FLL based on dual-frequency resonance tracking (DFRT). Instead of relying on a fixed phase condition, this solution probes the resonator with two frequencies in order to digitally locate the amplitude peak. This approach was reported to decouple the clock operating point from the phase delays introduced by the sustaining electronics [67].

The supply voltage affects a quartz oscillator mainly through the sustaining and compensation electronics rather than through the elastic properties of the resonator itself. Variations in $V_{DD}$ can shift amplifier bias, loop phase, resonator drive level, semiconductor capacitances, and varactor control voltage, producing frequency pushing and converting supply noise into phase noise through phase modulation and amplitude–frequency coupling [13,15,132]. A representative supply-voltage sensitivity for a high-quality crystal oscillator is $5\times 10^{-11}/V$ [15]; the value remains architecture- and condition-dependent [13].

Supply-induced errors are reduced using low-noise voltage regulation, filtering, stable references, and amplitude or drive-level control [27,133]. Representative implementations limited the frequency error to ±1 ppb for a ±5% supply variation in a miniature OCXO [42], below 5 ppt in a regulated industrial OCXO [40], and ±0.16 ppm for a ±10% change in $V_{DD}$ in a digitally controlled TCXO [130]. In low-power and analog TCXOs, stable regulation of the sustaining and compensation circuits is especially important because reduced voltage headroom increases their sensitivity to supply variations [36,129].

In MEMS oscillators, the influence of the power supply voltage is more direct than in quartz oscillators, particularly when the resonator relies on electrostatic actuation or capacitive displacement transduction. The IC supply voltage also affects the sustaining amplifier, transistor noise, bias network, converters, amplitude-control loops, and any synchronization loops. However, the DC polarization voltage applied directly to the resonator introduces an additional mechanism affecting frequency stability. In electrostatic structures, the DC bias voltage changes the force between the electrodes and introduces a negative stiffness component, known as electrostatic spring softening. The effective resonator stiffness then decreases relative to the purely mechanical stiffness. Because the resonant frequency depends on the stiffness-to-mass ratio, even minor fluctuations in the bias voltage translate directly into frequency shifts [68,117]. Analogous to load capacitance modulation in a TCXO, tuning the bias voltage in MEMS architectures can serve as a stabilization method. If the bias voltage is noisy, thermally unstable, or dependent on the aging of the reference circuits, it introduces additional drift and phase noise. Conversely, when precisely controlled, it provides an electronic tuning mechanism for frequency trimming and real-time compensation of environmental disturbances [17,57,134]. Using the voltage for compensation does not eliminate the prerequisite of power supply stability. Rather, it highlights the necessity of considering the entire power delivery network when designing systems that integrate MEMS oscillators. Consequently, voltage noise in MEMS devices can transduce into frequency error. Rigorous power filtering and low-noise supply sources are therefore essential to enhance overall frequency stability.

A summary of the effects of the power supply, load, and loop electronics on quartz and MEMS oscillator stability is given in Table 8.

In the architectures reviewed here, the sustaining and control electronics can dominate the residual electrical error once the intrinsic resonator sensitivity has been reduced. In quartz oscillators, the principal electrical contributions include load pulling, drive-level dependence, varactor control, and loop-phase variations, whereas MEMS oscillators additionally exhibit bias-dependent stiffness and electrical feedthrough. Regulation, filtering, circuit optimization, and digital correction can substantially reduce these sensitivities. A regulated quartz OCXO architecture has demonstrated a supply-induced error below 5 ppt for a 5% supply variation [40], but such improvements generally come at the cost of increased power consumption, start-up time, circuit area, or calibration effort. Supply excursion, output load, control voltage, and measurement bandwidth should therefore accompany reported stability results to enable meaningful comparison between oscillator implementations.

## 8. Radiation, Electromagnetic Fields, and Other Factors

### 8.1. Magnetic- and Electric-Fields Effects and Electromagnetic Interference

Quartz is diamagnetic and therefore has very low intrinsic magnetic susceptibility. However, a complete quartz oscillator can exhibit magnetic-field sensitivity because of magnetic materials in the mounting structure, package, and electronics. In particular, magnetostriction and field-induced changes in the elastic modulus of ferromagnetic supports can modify the stress transmitted to the quartz plate, shifting its resonance frequency through the force–frequency effect [13,136]. One of the primary mechanisms is the change of the Young’s modulus of the elastic support material in a magnetic field, which alters the stress distribution within the quartz plate and, consequently, shifts the resonant frequency via the force-frequency effect [136]. Measurements of oscillators used in satellite instrumentation have reported sensitivities on the order of (10 ppt/G) have been observed, with both magnitude and sign depending on the resonator design, mounting materials, and field orientation [137]. Consequently, in high-precision oscillators, design considerations encompass not only the resonator itself, but also material selection and magnetic shielding of the enclosure.

The influence of magnetic fields on the frequency stability of standard MEMS oscillators is usually considered marginal. Nevertheless, ferromagnetic materials present in packaging components can induce an indirect effect. The deposition of an additional magnetostrictive thin film in piezoelectric MEMS devices changes the influence of the magnetic field on the oscillator by altering the effective Young’s modulus. This configuration induces a measurable frequency shift. Such implementations achieve a high sensitivity of 28.5 Hz/nT [138], which is why they are deployed as precise magnetic-field sensors.

Time-varying electromagnetic fields can couple into PCB traces, ground loops, and supply lines, causing electromagnetic interference (EMI) that produces amplitude, phase, thermal, or bias disturbances. Furthermore, transient electromagnetic pulses can induce unwanted currents within conductive enclosures and circuit boards. Immunity therefore depends on proper grounding, shielding, supply filtering, and PCB layout [27,99,133]. In crystal oscillators, implementing a differential topology with dynamic phase injection has been demonstrated to attenuate EMI-induced phase noise by 10–15 dB without additional shielding [133]. For MEMS OCMO architectures, EMI mitigation instead relies on shielding, precise grounding, supply filtering, and layout isolation, together with the radiation and EMI robustness of the integrated control electronics.

A static DC electric field, in turn, can interact directly with the resonator via the electroelastic effect. This sensitivity depends on the crystal cut angle. AT-cut resonators show negligible sensitivity to this effect. Conversely, in SC-cut resonators, often used in precision OCXOs, the influence of the electric field can be measurable [13]. Furthermore, prolonged exposure to a static electric field induces the electromigration of ionic impurities within the crystal. This mechanism manifests directly as frequency drift and severely degrades long-term stability [99].

A summary of the effects of magnetic and electric fields and EMI is given in Table 9.

### 8.2. Ionizing Radiation

Ionizing radiation affects quartz oscillators in transient and permanent ways, depending on the radiation type, accumulated dose, and dose rate. In the case of pulsed X-ray or gamma radiation, electron-hole pairs are generated within the crystal. These carriers subsequently interact with structural defects and chemical impurities within the quartz lattice. The resulting transient increase in motional resistance and reduction in the resonator quality factor *Q* can disturb the loop gain, oscillation amplitude, and phase. Pulsed-irradiation experiments reported a pronounced but short-lived increase in series resistance after doses on the order of $10^{4}\;\mathrm{rad}$, even when the residual frequency shift was small [139,140].

Persistent frequency shifts arise mainly from radiation-induced changes in the charge state and distribution of impurity-related defects, particularly aluminium centers compensated by mobile alkali ions. These changes locally modify the elastic properties of quartz and therefore its resonance frequency [141]. Due to its higher background impurity concentration, natural quartz can exhibit frequency shifts on the order of several to tens of ppm following high accumulated doses. Using synthetic quartz limits this effect, and additional purification by annealing and electrodiffusion in a strong electric field (sweeping) reduces the alkali-ion concentration and yields a much lower radiation sensitivity [13]. In the tested commercial TCXO modules used in satellite instrumentation, the permanent radiation-induced frequency shift was approximately linear. Although this phenomenon is in itself an oscillator error, it makes it possible to turn the device into an onboard hardware dosimeter capable of estimating the total absorbed ionizing dose with an error below 8% [142]. Therefore, the radiation hardness of a quartz oscillator is not determined solely by the electronic circuit, but also depends on the material, resonator processing technology, crystal cut, impurity level, and design of the oscillation loop.

In MEMS oscillators and resonators, the radiation mechanisms differ from those in quartz, because the resonant structure is typically silicon, piezoelectric thin films, or composite multilayers. In many silicon MEMS devices exposed primarily to total ionizing dose (TID), the bulk mechanical structure may remain functional, while degradation is dominated by charge trapping in dielectrics and at material interfaces. In electrostatically transduced devices, the resulting parasitic electrostatic forces can shift the bias point and resonance frequency, modify the pull-in voltage, or cause stiction. Therefore, no universal radiation-dose threshold can be assigned to all MEMS topologies.

Piezoresistive and surface-dominated resonators may additionally exhibit radiation-induced changes in surface charge, carrier concentration, stress, and effective elastic properties. Electrostatic systems with exposed dielectrics are highly sensitive to ionizing radiation. The accumulation of trapped charge within these layers generates parasitic electrostatic forces that can detune or short the movable electrodes. Depending on the specific geometry and shielding, radiation-induced degradation in unshielded electrostatic MEMS has been reported at accumulated doses ranging from a few krad to over 100 krad [143]. Ionizing radiation can also generate charge in the surface oxide, change the carrier concentration in silicon bulk, and modify the elastic properties of the material. In piezoresistive MEMS devices irradiated with 10 keV X-rays, a decrease of the resonant frequency by about 25 ppm was observed at an accumulated dose of 2.1 Mrad. Ionizing radiation generates near-surface trapped charges. This process can depassivate hydrogen atoms previously bound to dopant impurities, thereby increasing the carrier concentration. The Young’s modulus also decreases and, consequently, the frequency drops [144]. Under heavy-particle irradiation, ionization, charge trapping, and crystal-lattice damage occur simultaneously. For silicon MEMS resonators exposed to a proton beam, the direction of the frequency shift can depend on the particle energy. When ionization effects dominate, the frequency decreases, whereas when displacement damage dominates, the material can effectively stiffen and the frequency increases [145]. The radiation response of a complete MEMS oscillator must consequently be evaluated separately for the resonator material, dielectric and surface layers, transduction electrodes, bias network, and sustaining and control electronics.

A summary of the effects of ionizing radiation is given in Table 10.

Radiation sensitivity is strongly dependent on oscillator technology, architecture, and operating conditions. In quartz oscillators, the dominant mechanisms are associated mainly with impurity-related defect processes in the crystal [139,140,141] and radiation-induced perturbations of the sustaining electronics, whereas MEMS devices additionally exhibit charge trapping in dielectric layers and interfaces, surface-related effects, displacement damage, and degradation of integrated control electronics [144,145]. Radiation sensitivity can be reduced through high-purity swept quartz, radiation-tolerant materials and interfaces, shielding, appropriate transduction schemes, and radiation-hardened electronics, but these measures impose additional material, process, circuit, and packaging constraints. Reported sensitivities illustrate this dependence rather than a consistent achievable-improvement figure: a commercial quartz TCXO showed a dose-estimation error of ±8% to ±20% above 20 Gy depending on the model [142], while a MEMS cantilever showed a resonance-frequency decrease of about 25 ppm at an accumulated dose of 2.1 Mrad [144]. The resulting frequency response nevertheless remains dependent on radiation type, particle energy, accumulated dose, dose rate, device bias, and temperature, which limits the transferability of results between devices and test conditions. A major remaining challenge is the system-level qualification of complete MEMS oscillators under conditions directly comparable with established quartz devices, since much of the available MEMS evidence still concerns resonators or sensors rather than fully integrated timing oscillators.

## 9. Discussion

Temperature remains the most extensively characterized environmental factor affecting oscillator frequency stability. For quartz oscillators, the mechanisms of frequency change and the limits of achievable stability have been studied for decades [14,15]. The conventional division into XO, TCXO, and OCXO allows designers to quickly match the oscillator class to specific application requirements, although all three architectures continue to evolve. In modern TCXOs, the residual error over the industrial temperature range has been reduced to fractions of a ppm, but extending the operating range requires increasingly complex correction schemes [35,37]. In OCXOs, operation of an SC-cut crystal near the turnover point of its static f(T) characteristic, combined with segmented compensation and higher-order polynomial correction, enables miniature designs to achieve single-ppb frequency stability [38,41], while selected laboratory implementations have reported ppt-level performance [42]. At this level, stability is increasingly limited by thermal dynamics rather than by the static characteristic alone. For example, achieving an ADEV on the order of $10^{-13}$ at 100 s may require ambient-temperature fluctuations to remain below 10 mK under the reported test conditions [96]. Power cycling also introduces frequency retrace, which contributes to long-term instability [101].

Research on MEMS oscillators is more recent, but rapid progress has been reported in temperature compensation and system-level integration. The temperature coefficient of frequency was reduced through material selection and geometric design [21], then through degenerate doping tuned to the crystallographic axis and the proportions of the structure [49]. Most recently, advanced mode engineering has been implemented. Nonlinear mode coupling within the BSE scheme has been demonstrated at the resonator level to achieve a stability of 1.5 ppb at 1000 s without thermal control power [74]. With the integration of local temperature sensing and closed-loop regulation, oscillator stability reaches the ppb range [62]. Furthermore, a two-stage off-chip temperature-compensation scheme based on fuzzy PID control held the oscillator at 35 °C to within ±1 mK while the surrounding chamber was at −10 °C, achieving a frequency stability of ±0.063 ppm [66]. Building upon this approach, a related system from the same group pairs fuzzy PID thermal stabilization with active nonlinear amplitude–frequency feedback operating within the Duffing regime, suppressing residual thermal drift down to ±14 ppb [69]. At high temperatures, resonators fabricated from wide-bandgap materials, such as silicon carbide and aluminum nitride, readily outperform conventional silicon resonators [18]. However, what makes MEMS particularly attractive is the ability to integrate the resonator, sensor, electronics, and compensation circuitry into a single compact and mechanically robust module.

Acceleration sensitivity is also well understood. In quartz resonators, it is described by the vector Γ. Theory has long explained how acceleration turns into frequency modulation [118]. Typical miniature AT-cut resonators exhibit sensitivities of a few ppb/g. This value can be lowered by pairing crystals with opposing sensitivity vectors (below 0.1 ppb/g [111]), by choosing the electrode geometry relative to the anchoring (about 0.5 ppb/g [114]), or by operating on the third overtone (about 0.3 ppb/g [115]). In OCXOs, it can be additionally reduced by active accelerometer-based correction [116]. This reduction cannot be pushed indefinitely. In a resonator with nulled first-order sensitivity, the nonlinear second-order effects leave shifts on the order of $10^{-11}$/g [112].

MEMS oscillators generally tolerate shock and vibration well because of their small moving mass. However, their package, anchors, and isolation structures can still limit performance. By utilizing a central anchor, the out-of-plane acceleration sensitivity was reduced to 0.74 ppb/g, while extreme drop tests revealed a maximum transient frequency deviation of −7.7 ppm measured 1.7 ms after an 11,000 g impact. Notably, the oscillator continued to operate nominally even after experiencing eight shocks at 5000 g and four at 11,000 g [117]. Furthermore, vibration may therefore require motion limiters and stress-wave-absorbing packaging [45]. Pressure demands similar attention. In subsea systems, oil or resin transfers the load directly onto the component package. Consequently, a factory-sealed oscillator can deform, change frequency, or fail. In a hydrostatic test of four commercial quartz oscillators, the output frequency became more variable at 400 bar and all four units failed completely at 425 bar, with significant buckling of the casing documented for the 8 MHz device [123]. MEMS oscillators, in contrast, showed a stronger negative frequency trend in the cited tests and may require differential compensation or package-level mitigation [124,127].

The operating environment also includes radiation and electromagnetic fields. In quartz oscillators, ionizing radiation produces both transient disturbances and permanent frequency changes associated with point defects [140,141]. Radiation sensitivity can be reduced by using high-purity synthetic quartz processed by electrodiffusion to lower the concentration of mobile alkali ions [13]. The strongly linear dependence of the frequency shift on dose, in turn, allows off-the-shelf TCXO modules to be used as onboard hardware dosimeters [142]. In many silicon MEMS devices, the bulk silicon structure can remain largely intact at mission-relevant doses, while failures are often dominated by trapped charge in dielectric layers and by the radiation sensitivity of the readout and control electronics. The problem is mitigated with grounded internal shields and with designs that avoid electrostatic transduction [143,145]. Because radiation-hardened CMOS is a well-developed technology, pairing the resonator with a separately selected radiation-tolerant ASIC rather than integrating both monolithically could concentrate the remaining hardening effort on the MEMS structure itself; this architecture is recommended for radiation-tolerant MEMS sensors and is an engineering inference for timing devices rather than a demonstrated oscillator result [143].

Control methods and machine learning are playing an increasingly important role in reducing environmentally induced frequency errors. In TCXOs, the thermal hysteresis that for years limited compensation effectiveness is modeled first as an f(T,H) dependence on the temperature history, which reduced the error from about 700 ppb to 150 ppb [32]. Neural-network approaches, including recurrent LSTM models, have further reduced dynamic compensation errors to the sub-ppm range [100,102]. Recent reviews also discuss control techniques addressing temperature compensation, drive-level dependence, and phase-noise reduction in quartz oscillators [135]. In MEMS oscillators, advanced mode engineering enables a single structure to function simultaneously as a physical-quantity sensor and a stable frequency reference [74].

From an application perspective, the relative importance of these environmental effects depends on both device sensitivity and the magnitude of the disturbance encountered during operation. Temperature generally dominates uncompensated oscillators, but once thermal errors are reduced to the sub-ppm or ppb range, other contributions can become comparable or larger. For example, an acceleration sensitivity of several ppb/g can produce tens of ppb of frequency shift in a dynamic environment, while aging accumulates with operating time and radiation, pressure, or shock may dominate under severe exposure. Similarly, aging typically contributes on the order of 0.1–1 ppb/day in compensated devices, while supply, loading, and electronic effects individually contribute sub-ppb to few-ppb fractional frequency shifts under regulated operation. The hierarchy of environmental contributions is therefore application dependent, and oscillator performance should be evaluated through an error budget constructed for the intended operating profile rather than from isolated sensitivity values [11,27,31]. For a first-order environmental error budget, each contribution may be estimated from the corresponding frequency-sensitivity coefficient and the expected variation of the relevant parameter, i.e., $y_{i}≃S_{i}\Delta x_{i}$. Independent random contributions may be combined by root-sum-square, whereas correlated contributions require covariance terms. In practice, independence should not automatically be assumed, since temperature, self-heating, supply, loading, and electronic effects may be mutually coupled.

The main research gap remains the assessment of combined environmental effects and the long-term comparison of MEMS and quartz oscillators under identical operating conditions. Standardized procedures for measuring the environmental sensitivities of frequency generators already exist [27]. In the comparative studies reviewed here, these procedures have not been applied consistently across the two technologies. Comparative studies have been published, but those reviewed here adopt different device samples, environmental subsets, and reporting conventions, so their results cannot be pooled directly. Even the side-by-side measurement discussed in Section 4 [109] covered no mechanical, pressure, field, or radiation environment. Although quartz aging has been studied for decades, its magnitude remains strongly design-dependent. Results for MEMS oscillators are promising, but many studies focus on demonstrators or relatively short observation periods. Open data sets covering months and years of operation are needed, with simultaneous recording of temperature, pressure, vibration, supply, and frequency. Such data would help determine when the integration of multifunctionality of MEMS outweighs the proven aging stability of quartz.

Quartz OCXOs remain well suited to synchronization, precision timekeeping, and applications requiring low noise over long averaging times [135]. MEMS oscillators may offer advantages when size, mass, shock resistance, low acceleration sensitivity, and integration with electronics are the primary requirements, which is particularly important in sensing applications [146]. The preferred technology therefore depends on which performance attributes are most critical in the intended application.

## 10. Conclusions

This review has compared the mechanisms that degrade the short- and long-term frequency stability of quartz and MEMS oscillators and the compensation and mitigation methods available for both technologies. In contrast to reviews centered mainly on MEMS resonators or thermal compensation, its principal contribution is a common cause-based classification of thermal, mechanical, electrical, environmental, and radiation-induced effects, including their origins in the resonator, package, sustaining electronics, and control system. The framework connects these mechanisms with sensor-system errors, an operating-profile assessment of environmental contributions, and the condition-preserving comparison in Appendix A. Quartz oscillators retain an advantage in established long-term stability and low-noise performance, whereas MEMS devices offer compactness, mechanical robustness, multifunctionality, and integration with sensing and control electronics.

No oscillator technology is optimal for all applications. Selection should therefore be based on an explicit error budget covering the dominant environmental variables and the relevant observation time. Short-term performance is commonly affected by resonator and electronic noise, supply disturbances, vibration, and thermal fluctuations, whereas long-term stability is increasingly governed by aging, retrace, package stress, and slowly varying environmental conditions. A key research gap is not the absence of measurement methods but the lack of their systematic application to comparison across the two technologies. Open datasets collected over months or years would allow the integration advantages of MEMS to be evaluated objectively against the well-established aging performance of quartz.

## Figures and Tables

**Table 1:** Terminology used for oscillator frequency performance. The indicated time scales are representative rather than strict boundaries and depend on the oscillator type, nominal frequency, measurement bandwidth, observation duration, and application.

Quantity	Physical Meaning	Representative Metric	Relevant Time Scale
**Frequency accuracy**	Closeness of the measured or mean oscillator frequency to the nominal frequency $f_{0}$ or to a specified reference under stated operating and measurement conditions.	Absolute frequency error $\Delta f=\bar{f}-f_{0}$ in Hz, or mean fractional frequency offset $\bar{y}=\left(\bar{f}-f_{0}\right)/f_{0}$, commonly expressed in ppm, ppb, or ppt [12].	No intrinsic time scale. The measurement interval, warm-up state, reference, and operating conditions must be stated.
**Frequency stability**	Ability of the oscillator to maintain a constant frequency over time under specified conditions. It characterizes temporal frequency fluctuations instead of the absolute offset from $f_{0}$.	ADEV $\sigma_{y}\left(\tau \right)$, MDEV, HDEV, PDEV, or another stated stability statistic as a function of averaging time τ; dimensionless fractional frequency [11,25].	Explicitly dependent on τ; commonly evaluated from sub-second intervals to hours or days, depending on the application and data record.
**Drift**	Slow, systematic frequency change with time, caused by environmental variations, operating conditions, or electronic parameter changes, as well as by internal processes such as stress relaxation; the latter is treated separately as aging when it occurs under nominally constant conditions (see below)	Slope dy/dt, fitted trend, or cumulative fractional frequency change; for example, ppb/h, ppb/day, or ppm/year [27].	Typically minutes or hours to months. The observation interval and environmental conditions must be stated.
**Aging**	Long-term frequency change caused by time-dependent internal physical or chemical processes in the resonator, package, mounting structure, or sustaining electronics under nominally constant conditions.	Aging rate or cumulative aging, commonly expressed in ppb/day, ppm/year, or fractional frequency change over a specified interval [27,28].	Days to years, normally after a defined warm-up or stabilization period and under nominally constant environmental and operating conditions.
**Phase noise**	Frequency-domain description of random phase fluctuations φ(t) of the oscillator signal. It characterizes the spectral distribution of phase fluctuations around the carrier.	Phase-fluctuation power spectral density $S_{\varphi }\left(f\right)$ in ${\mathrm{rad}}^{2}$/Hz, or the single-sideband quantity $L\left(f\right)=\frac{1}{2}S_{\varphi }\left(f\right)$ in dBc/Hz, which is the form used in oscillator literature, data sheets, and commercial specifications [11,12].	Defined against Fourier offset frequency *f* rather than a single averaging time; low offsets correspond to long observation intervals and high offsets to short ones.
**Jitter**	Time-domain variation of clock-edge or zero-crossing positions relative to their ideal times. It describes timing uncertainty caused by phase fluctuations.	RMS time jitter $\sigma_{t}$, peak-to-peak jitter, period jitter, cycle-to-cycle jitter, or time-interval error, commonly expressed in s, ps, or ns; the phase jitter deviation is obtained by integrating $S_{\varphi }\left(f\right)$ between stated cutoff frequencies, so the integration bandwidth must always be specified [11].	From individual oscillation cycles to the stated observation interval; strongly dependent on the integration bandwidth and jitter definition.

**Table 2:** Reported temperature-stability results for quartz and MEMS oscillators.

Approach	Reported Metric	Range Averaging Time	Thermal Control and Power	Evaluation Level	Refs.
**Quartz oscillators**					
Uncompensated AT-cut XO	Total frequency variation of tens of ppm	−40 to 85 °C	None	Oscillator; literature and data-sheet values	[15]
TCXO, closed compensation loop, 100 MHz	Frequency stability within ±0.22 ppm	−40 to 85 °C	None (no oven)	Oscillator, laboratory prototype	[35]
TCXO, analog correction with higher-order multiplier	Frequency stability within ±0.8 ppm (simulated)	−40 to 95 °C	None (no oven)	Post-layout simulation in Cadence Virtuoso; chip being fabricated in SMIC 0.13 μm	[36]
TCXO, time-domain accuracy enhancement	Frequency stability within ±1.4 ppm	−55 to 115 °C	None (no oven)	Oscillator, laboratory prototype	[37]
Miniature OCXO with integrated heater	Frequency stability within ±3 ppb	−40 to 85 °C	Oven; power not stated	Oscillator, laboratory prototype	[38]
OCXO in 5×3.2 mm package	Frequency stability within ±20 ppb	−40 to 95 °C	Oven; power not stated	Oscillator, laboratory prototype	[39]
OCXO with segmented polynomial compensation	Error of the most improved unit reduced from ±4.29 to ±0.153 ppb	−40 to 85 °C	Oven; 20×20 mm design	Oscillator, laboratory prototype	[41]
Miniature OCXO with fourth-order temperature control	ppt level (value not resolved further)	−40 to 85 °C	Oven with higher-order control	Oscillator, laboratory prototype	[42]
Complete OCXO with an external thermoelectric cooler	Warm-up shortened from ≈3 min to <1 min	Warm-up transient	External TEC and thermoresistor driven by a separate control module, added to a complete OCXO	Commercial OCXO on a test PCB	[44]
**MEMS resonators, passive compensation**					
${\mathrm{Si/SiO}}_{2}$ composite, oxide surface film	Total frequency variation <200 ppm	−40 to 125 °C	None	Resonator, wafer-scale encapsulated	[46]
${\mathrm{Si/SiO}}_{2}$ composite, characterized	$TCF_{1}=0$; TCF2≈−20 ppb/∘C2; <2 ppm drift; *Q* increased by 15–50%	Turnover tunable −55 to 125 °C; drift over >6 months	None	Resonator; dielectric charging in oxide noted	[47]
SilOx composite, embedded oxide pillar matrix	Overall frequency drift 83 ppm; improved *Q* stability; no *Q* or insertion-loss penalty	−40 to 80 °C	None	Resonator	[48]
Degenerate doping, elastic-constant characterization	$TCF_{1}$ of $c_{11}-c_{12}$ zero at $\approx 2\times 10^{19}$ cm^−3^	−40 to 85 °C	None	Material characterization from resonator arrays	[50]
Degenerate doping, design rules	Design-rule framework, no single figure of merit	−40 to 85 °C	None	Resonator design methodology	[51]
Degenerate doping, extensional mode	Turnover point predicted near room temperature at 0.5 and $1\times 10^{19}$ cm^−3^	Turnover position	None	Analytical and FEM study, not a measurement	[49]
Degenerate doping, TPoS extensional mode	Overall frequency variation <245 ppm	−40 to 85 °C	None	Resonator, ≈25 MHz	[52]
Doping and crystal orientation, bulk capacitive modes	Measured frequency shift ±16 ppm (square-extensional); ±20 ppm (length-extensional)	−40 to 85 °C	None	Resonator, $22.5^{\circ }$ from 〈110〉	[53]
Doping, orientation and oxide composite, ScAlN-on-Si	Frequency variation ±21.5 ppm; $Q_{un}=10,017$	−40 to 85 °C	None	Resonator, 24.44 MHz	[54,55]
Geometry engineering, folded anchors	TCF reduced by two orders of magnitude; 2500 ppm total shift; turnover at 27 °C	0 to 90 °C	None	Resonator, single-layer CMOS–MEMS	[56]
Geometry engineering, modal design	TCF1=−0.39 ppm/∘C; TCF2=−7.3 ppb/∘C2; total deviation 38 ppm	−20 to 70 °C	None	Resonator, pseudo-Lamé	[57]
Mechanical coupling, TCF manipulation	Measured frequency shift ±10 ppm	−40 to 85 °C	None	Resonator, 18 MHz coupled square-extensional mode	[58]
Mechanical coupling, turnover-point adjustment	Turnover point moved above the industrial range	Turnover position	None; enables subsequent micro-oven control	Resonator, breathing-ring coupled to length- and width-extensional modes	[59]
Mechanical coupling, simultaneous *Q* and TCF improvement	Frequency shift ±55 ppm; best device $Q_{un}=80,695$; population averages give a 2.44× *Q* gain and 2.5× lower drift	−40 to 85 °C	None	Resonator, 25.202 MHz	[60]
**MEMS resonators, passive modal and operating-point selection**					
Curved-beam piezoelectric resonator, beat frequency of two modes	ADEV 127 ppb	τ=1.45 s, during a continuous 25 to 75 °C change	None; requires excitation of two modes and beat-frequency processing	Resonator	[90]
Curved-beam piezoelectric resonator, temperature-insensitive extremum	ADEV 2.0 ppb	τ=2.51 s, held near 55 °C	Passive resonator design, but external temperature control during evaluation; power not stated	Resonator	[90]
**MEMS oscillators, active electronic compensation**					
Interpolated-DFT frequency estimation with PLL-based calibration	Output frequency stable within ±0.3 ppm; discrepancy between measured and actual frequency < ±0.4 Hz after correction	−40 to 85 °C	No heating; power not stated	49.5 and 50 MHz oscillators with calibration module; simulation and experiment	[61]
**MEMS oscillators, micro-oven control (OCMO)**					
Dual Lamé-mode ovenized resonator, ambient-temperature ramp	Averaged frequency stability ±1.5 ppb	Ambient ramped −40 to 60 °C over 10 h	Micro-oven, <30 mW	Resonator in epi-seal platform	[20]
Dual Lamé-mode ovenized resonator, steady-state test	Average change ≈ ±0.5 ppb; minimum ADEV <0.65 ppb at τ=1000 s	One week at a constant ambient of 20 °C	Micro-oven, 12 mW for the Lamé mode	Resonator in epi-seal platform	[20]
Oven-controlled dual-resonator platform, ScAlN on doped Si	Real-time frequency stability ±100 ppb	−40 to 85 °C	Micro-oven; maximum 40 mW heating power	Resonator platform with control loop	[62]
Micro-oven-controlled dual-mode piezoelectric resonator	Real-time frequency stability ±190 ppb	−40 to 105 °C	Micro-oven, PLL-based control; maximum 39 mW	Resonator	[63]
Dual-mode distributed Lamé resonator, on-chip ovenization	Sub-ppb stability	Ovenized at the 250 °C turnover point	On-chip micro-oven; integrated heating and sensing resistors	Resonator with oscillator loop	[64]
Micro-oven in wafer-level vacuum package	TCF≈0.6 ppm/∘C	−40 to 80 °C	Micro-oven with thermal isolation	Resonator in WLVP	[65]
Two-stage compensation with off-chip oven	Frequency stability within ±0.063 ppm	Range not stated	Off-chip oven; polyimide heating films, fuzzy PID control to ≈±1 m°C	Oscillator	[66]
**MEMS oscillators, linear active feedback and dual-mode thermometry**					
Dual-frequency resonance tracking (DFRT) in a frequency-locked loop	MDEV $\approx \;8\times 10^{-12}$; possible drift −4.1±2.5 ppt/day	τ=8 h; ambient laboratory conditions	Active regulation at the turnover point, a mode ratio serving as thermometer	268 MHz clock, encapsulated resonator	[67]
Single-mode Lamé resonator, macro-scale ovenization	MDEV 42 ppt; sub-ppb up to 4800 s	τ=85 s and τ≤4800 s	External macro-scale heating chuck in air; increased volume and power	Resonator with sustaining oscillator loop	[68]
**MEMS oscillators, nonlinear and mode-coupling compensation (laboratory demonstrations)**					
Nonlinearity-mediated drift suppression after first-stage oven	Frequency stability ±14 ppb	During transient ambient-temperature changes, loop active	Oven plus Duffing operation; drive-level dependent	Resonator	[69]
Duffing-regime silicon oscillator	Frequency stability ±1.88 ppb	Within ±0.735 °C of the 56 °C inflection point (total 1.47 °C window)	Active; narrow drive and temperature window	Resonator	[70]
Internal resonance, 1:2 mode coupling in a stepped beam	Frequency stability improved more than twentyfold	Not stated	No oven; requires a near-integer modal frequency ratio	Resonator	[72]
Blue-sideband excitation, sum-frequency reference	ADEV 1.5 ppb; 11.9 ppb under combined temperature and electrostatic disturbance	τ=1000 s	No oven; requires prescribed modal frequency ratio	Resonator	[74]
Adaptive mode-coupling stabilization	≈700× improvement at τ=1000 s; >1000× at τ=802 s under varying temperature	τ=802–1000 s	Phase control; no oven	Coupled resonator pair	[91]
**Commercial MEMS oscillators: manufacturer specifications (guaranteed limits and typical values)**					
SiT5811 holdover OCXO	Frequency stability ±1 ppb (guaranteed); frequency slope ±0.01 ppb/∘C (typical)	−40 to 95 °C	Internal temperature stabilization; 420 mW typical at 3.3 V	Product specification	[87]
SiT5812 holdover OCXO	Frequency stability ±1 ppb (guaranteed); frequency slope ±0.01 ppb/∘C (typical)	−40 to 95 °C	Internal temperature stabilization; 460 mW typical at 3.3 V	Product specification	[92]
SiT5543 ruggedized Super-TCXO	Frequency stability ±5 ppb (guaranteed)	−40 to 95 °C (widest option offered)	Electronic compensation, no oven; 44 mA typical at 3.3 V (≈145 mW), 19.2 MHz, no load	Product specification	[89]

**Table 3:** Summary of the effects of thermal dynamics on quartz and MEMS oscillator stability.

Effects	Compensation and Mitigation
**XO**	
Nonuniform temperature distributions and internal thermal gradients, arising from either environmental transients or localized heating, causing deviations from the isothermal frequency response [43,93] Local dynamic gradients with an influence comparable to or greater than that of the mean temperature shift [43]	Dual-parameter correction based on the mean temperature and spatial gradients, reducing the residual error to less than 5% of the initial disturbance [94]
**TCXO**	
Local sensor temperature differing from that of the active resonator region [99] Dynamic frequency-tracking errors during rapid thermal transients	Dynamic state-dependent f(T,t) modeling using artificial neural networks [100]
**OCXO**	
Frequency shifts caused by nonuniform oven heat flux, convective package cooling, and ambient-temperature fluctuations (achieving an ADEV of $10^{-13}$ at 100 s required fluctuations below 10 mK under the reported conditions [43,96]) Phase lag in the thermal feedback loop	Evaluation under dynamic thermal profiles representative of field operation [95] Thermomechanical optimization combined with a 52 h GNSS-disciplined aging-learning phase yielding a holdover time error below 1.5 μs over 24 h during a 40 °C cycle with a 1 °C/min gradient [95] Simultaneous environmental-chamber stabilization of temperature, humidity, and pressure (±25 mK, ±0.1% RH, ±12 Pa), improving USO stability by two orders of magnitude to $2\times 10^{-13}$ at 1–10^5^ s [97]
**MEMS oscillators with passive compensation**	
Miniaturized structures remaining sensitive to package- and anchor-induced stresses [18]	Biasing at the thermal turnover point (55 °C) yielding 2.0 ppb stability [90] Mode selection in a curved-beam piezoelectric resonator maintaining 127 ppb stability during a continuous 25–75 °C thermal ramp [90]
**MEMS oscillators with active compensation (OCMO)**	
Residual frequency drift under time-varying thermal conditions when passive thermal isolation is insufficient [91]	WLVP with an integrated micro-oven achieving $0.6\;\mathrm{ppm}{/}^{\circ }C$ [65] Adaptive closed-loop mode coupling improving frequency stability by more than a factor of 1000 [91] Closed-loop thermal regulation achieving 42 ppt stability at 85 s and sub-ppb stability up to 4800 s [68]

**Table 4:** Summary of the effects of thermal hysteresis and retrace on quartz and MEMS oscillator stability.

Effects	Compensation and Mitigation
**XO**	
Thermal-memory dependence of output frequency [13,23] Mechanisms including interfacial stresses, impurity migration, point defects, and circuit hysteresis [23] Apparent hysteresis caused by transient thermal gradients [23]	Characterization of hysteresis and retrace according to IEEE Std 1193 [27] Using the resonator as its own thermometer (e.g., via the SC-cut B-mode) to eliminate probe-to-crystal temperature errors [99]
**TCXO**	
Static f(T) compensation assuming a unique frequency value for each temperature Residual hysteresis errors reaching hundreds of ppb [32]	State-dependent f(T,H) modeling reducing residual error from 700 to 150 ppb [32] LSTM prediction of combined temperature and hysteresis effects with an RMSE below 0.05 ppm [102] Artificial neural networks modeling dynamic, history-dependent characteristics [100]
**OCXO**	
Frequency retrace after power-off, cooling, and reheating to the nominal operating temperature [101] Natural frequency recovery to within ±0.2 ppb of the pre-shutdown value about 10 h after restart [101]	Thermomechanical optimization limiting hysteresis to below $\pm 5\times 10^{-11}$ under a 1 °C/min thermal gradient [95]
**MEMS oscillators with passive compensation**	
Thermal-memory magnitude strongly dependent on the anchoring architecture [104] Frequency and *Q* hysteresis in resonators requiring multiple anchor points, becoming more pronounced for shorter support beams and reaching a twenty-fold difference in *Q* between cooling and heating sweeps over 40–300 K [104] Progressive reduction of hysteresis during successive thermal cycles [104]	A hermetically sealed structure with a single central anchor showing no measurable hysteresis over 460 cycles from −50 to 80 °C [103] Reduction or suppression of hysteresis through optimized anchor geometry that isolates the resonator from axial stress caused by differential expansion of the encapsulation layers [103]
**MEMS oscillators with active compensation (OCMO)**	
Excellent thermal coupling from integrating the resonator within its micro-oven on a single chip, in contrast to the large thermal hysteresis of quartz OCXOs caused by limited coupling to the heating element [63]	Dual-mode ratiometric stabilization using dual-frequency resonance tracking (DFRT) to suppress sensitivity to temperature-induced phase and gain variations in the electronics [67]

**Table 5:** Summary of aging and long-term drift mechanisms on quartz and MEMS oscillator stability.

Effects	Compensation and Mitigation
**XO**	
Adsorption and desorption of molecules on active resonator surfaces [28] Redistribution of impurities and changes in electrode mass and internal stress [28] Relaxation of thermomechanical stresses induced during cutting, polishing, and mounting [15] Long-term drift of sustaining-loop parameters, including capacitances, resistances, bias points, and drive levels [14]	High-vacuum hermetic sealing, strict surface cleanliness, and high-temperature baking [28] Elevated-temperature screening to accelerate aging mechanisms and predict long-term drift [28]
**TCXO**	
Oscillator aging compounded by drift in active and passive circuit components, including voltage references, amplifiers, resistors, and capacitors [14,27]	Kalman-filter estimation of temperature and aging drift (enabling a ±3 ppm TCXO to replace a ±0.01 ppm OCXO while maintaining timing error below 0.8 ms/day [108])
**OCXO**	
Continuous thermal stabilization restricts ambient volatility, making long-term drift highly systematic and predictable [28,106] Frequency retrace anomalies following power off and thermal cool-down [101]	Statistical batch-level aging models improving long-term stability by one to two orders of magnitude [106] Real-time phase-shift monitoring across the resonator terminals (enabling reference-free drift compensation and an aging rate of $9.8\times 10^{-12}$/day [107])
**MEMS oscillators with passive compensation**	
Small mass and high surface-to-volume ratio amplifying frequency detuning from molecular adsorption [17,18] Long-term thin-film instability, with film and film–oxide interface properties changing over time and the changes enhanced by temperature [28] Slow degradation of the cavity vacuum through gas leakage, increasing air damping and reducing *Q* over time [17,110]	WLVP isolating the resonator from atmospheric variations [21,110] Exponential quality factor *Q* degradation models for predicting hermetic-package lifetime and supporting reliability screening [110]
**MEMS oscillators with active compensation (OCMO)**	
Elevated micro-oven temperatures accelerating the aging of internal interfaces and electrode materials, a mechanism inferred from quartz-resonator and high-temperature MEMS studies rather than from a direct OCMO reliability test [18,28] Permanent changes in resonator mass, stress, and effective structural stiffness, again inferred for OCMO rather than directly measured [18,28]	Commercial holdover MEMS OCXOs with on-chip supply filtering and digital frequency tuning; specified aging of ±0.1 ppb per day under an 8 °C ITU-T temperature profile with 1 m/s airflow [87,92] Amplitude-tracking FLL with ratiometric stabilization (achieving an MDEV of $8\times 10^{-12}$ at an averaging time of 8 h and a drift of −4.1±2.5 ppt/day) [67]

**Table 6:** Summary of the effects of acceleration, vibration, and mechanical shock on quartz and MEMS oscillator stability.

Effects	Compensation and Mitigation
**XO**	
Fractional frequency shift caused by acceleration, described by $\Delta f/f_{0}=\Gamma \cdot a_{g}$ (typical sensitivity is about 2 ppb/g for miniature AT-cut resonators) [15,27,118] Frequency modulation and spectral sidebands caused by dynamic vibration [27,118] Mechanical excitation of the package and PCB caused by acoustic-pressure fluctuations [15] Force–frequency shifts caused by mounting stress and PCB flexure [113] Frequency shifts dependent on the force application point and resonator azimuthal orientation [113] Stability degradation in dynamic flight environments compared with laboratory conditions [4]	Electrode-geometry optimization relative to the anchor points, reducing acceleration sensitivity to 0.5 ppb/g [114] Third-overtone operation, reducing acceleration sensitivity to approximately 0.3 ppb/g [115] Pairing two crystals with antiparallel acceleration-sensitivity vectors, with reported sensitivities below 0.1 ppb/g over 10–40 MHz [111]
**TCXO**	
Residual second-order acceleration sensitivity on the order of $10^{-11}$/g after the first-order term is nulled, estimated for a symmetric Y-cut resonator [112]	Compensation using two crystals with antiparallel acceleration-sensitivity vectors, whose mechanical responses cancel (sensitivities below 0.1 ppb/g over 10–40 MHz have been reported [111])
**OCXO**	
Increased transmission of mechanical excitation due to the oven mass and thermal-insulation structures Frequency-stability degradation under dynamic operating conditions [4]	Real-time feedforward compensation using an integrated accelerometer and digital frequency correction [116]
**MEMS oscillators with passive compensation**	
Direction-dependent frequency shifts caused by acceleration (Lamé-mode resonators have shown sensitivities of 2.1 ppb/g in-plane and 3.8 ppb/g out-of-plane [117]) Transient frequency deviations during severe shocks (an 11,000-g shock produced a peak deviation of −7.7 ppm 1.7 ms after impact, with no degradation reported afterwards [117]) Irreversible frequency drift or device failure caused by vibration-induced structural fatigue [45]	Central-anchor design reducing out-of-plane acceleration sensitivity to 0.74 ppb/g [117] Motion-limiting structures and stress-wave-attenuating packaging reducing shock-induced damage and structural fatigue [45]
**MEMS oscillators with active compensation (OCMO)**	
Reduced mechanical stability of slender OCMO thermal-isolation suspensions [22]	Machine-learning error compensation demonstrated for MEMS inertial sensors under random vibration, where a bidirectional LSTM combined with a Kalman filter reduced the output standard deviation by 46.5% and 22.3% on two axes [45]

**Table 7:** Effects of humidity, pressure, and package atmosphere on quartz and MEMS oscillator stability.

Effects	Compensation and Mitigation
**XO**	
Moisture-induced changes in surface leakage currents and parasitic PCB capacitances [125] Moisture-induced frequency shifts in QCM sensors driven by either permittivity changes in a series humidity-sensitive capacitor [119] or mass loading of a graphene-oxide layer on the electrode [120] Transmission of hydrostatic pressure to the device package through oil or resin potting media [123] Pressure-induced frequency instability under hydrostatic high-pressure conditions, with thresholds strongly dependent on oscillator design [123] Package buckling and structural failure under extreme hydrostatic pressure; failures of commercial SMD packages were observed at 425 bar [123]	Integration into rigid titanium or steel pressure vessels to isolate the oscillator from extreme mechanical loads [123] Selection of oscillators and packages rated for the target pressure range [123]
**TCXO**	
Medium- and long-term frequency sensitivity to atmospheric humidity and possibly barometric pressure [125]	Enclosing the complete oscillator in a sealed container to hold ambient moisture and pressure approximately constant [125]
**OCXO**	
Changes in heat transport caused by the internal package atmosphere, including nitrogen, helium, or vacuum [16] Changes in warm-up time, thermal gradients, and steady-state heater power due to the internal gaseous medium [16] Residual frequency instability caused by ambient-pressure variations in ultra-high-stability measurement conditions [97]	Hermetically sealed ceramic packaging supporting thermal and frequency stabilization [126] Active stabilization of the measurement-chamber atmosphere to reduce environmentally induced frequency drift [97]
**MEMS oscillators**	
Pressure-dependent frequency shifts and residual offsets after hydrostatic pressure cycling [124] Temporary or permanent functional degradation during decompression under hydrostatic high-pressure testing [124] Residual-gas or helium ingress degrading the cavity vacuum, increasing damping, and reducing the resonator quality factor *Q* and oscillation margin [121,122] Package deformation and structural damage under severe cyclic pressure [124] Delamination risk in bonded multilayer structures due to coupled thermomechanical stresses [45]	Monitoring and lifetime prediction based on Q(t) degradation models for assessing wafer-level package hermeticity [110] Avoidance of helium-based leak testing or use of helium-resistant packaging to prevent cavity-vacuum degradation [121] Differential compensation using an integrated reference resonator to suppress common-mode environmental shifts [127] Matching of the temperature and pressure coefficients of the paired resonators to improve differential compensation [127]

**Table 8:** Effects of the power supply, electrical loading, and loop electronics on quartz and MEMS oscillator stability.

Effects	Compensation and Mitigation
**XO**	
Load-dependent frequency pulling governed by $\left(f_{L}-f_{q}\right)/f_{q}\approx C_{m}/\left[2\left(C_{0}+C_{L}\right)\right]$ [24,35] Frequency shifts caused by PCB parasitics, receiver impedance, and aging-induced changes in the effective load capacitance Supply-induced changes in amplifier bias, resonator drive level, semiconductor capacitances, and loop phase [13] Conversion of supply-voltage variations into frequency pushing and phase noise (the reported voltage sensitivity can be on the order of $5\times 10^{-11}/V$ [15])	Amplitude stabilization, local voltage regulation, and power-rail filtering [27]
**TCXO**	
Supply-induced shifts in the DC operating points of MOSFETs within the compensation network [36] Increased sensitivity of sustaining and compensation circuits under nanowatt power constraints [129]	LDO regulation and filtering of analog blocks [36] Digitally controlled amplitude-control loop, with a measured maximum frequency error of ±0.16 ppm under ±10% $V_{DD}$ variation [130] ΔΣ-modulated switching of a single load capacitance, with the complete TCXO consuming 43 nW at $25^{\circ }C$ [129]
**OCXO**	
Conversion of supply disturbances into phase noise [132] Phase modulation in the sustaining amplifier, amplitude–frequency coupling, and varactor-control noise [132] Nonlinear effects at excessive resonator drive levels [133]	Internal voltage regulation limiting residual frequency pushing to below 5 ppt for a 5% supply variation [40] Supply stabilization limiting the frequency error to ±1 ppb for a 3.3 V ±5% supply variation [42] Differential topology with dynamic phase injection, improving EMI immunity and start-up dynamics while reducing the crystal resonator drive current by up to 50% [133] Control-engineering approaches to temperature regulation, drive-level optimization, and phase-noise reduction, including system-level and FEM modeling [135]
**MEMS oscillators with passive compensation**	
Resonance-frequency shift caused by bias-induced electrostatic spring softening [68,117] Conversion of DC-bias fluctuations and drift into frequency error and phase noise Feedthrough capacitance altering the loop gain and phase, thereby increasing power consumption and sensitivity to electronic noise [131] Output loading increasing buffer rise time, switching current, and output jitter, with generally little direct effect on the nominal resonator frequency	Low-noise and thermally stable DC-bias references Reduction of feedthrough and parasitic capacitances [131]
**MEMS oscillators with active compensation**	
Frequency error and phase-noise degradation caused by supply-induced variations in amplifiers, bias circuits, converters, and control loops [17] Operating-point shifts caused by electronics phase delays in PLL-based architectures [67]	Temperature-dependent bias-voltage tuning compensating the drift of the mechanical spring constant [17,134] FLL based on DFRT, reducing sensitivity to electronics phase delays [67]

**Table 9:** Effects of magnetic and electric fields and EMI on quartz and MEMS oscillator stability.

Effects	Compensation and Mitigation
**XO**	
Magnetic-field-induced frequency shifts caused by ferromagnetic mounting and package components [13,136] Frequency shifts resulting from field-induced changes in the elastic properties of the supports and the associated force–frequency effect [136] Magnetic sensitivities on the order of 10 ppt/G, with magnitude and sign dependent on oscillator construction, mounting materials, and field orientation [137] Amplitude, phase, thermal, and bias disturbances caused by EMI coupling into PCB traces, ground loops, and supply lines [27,99,133]	Reduction or elimination of ferromagnetic materials and use of magnetic shielding [136] Careful grounding, supply filtering, shielding, and signal-path separation [27,133]
**TCXO**	
Conducted and radiated EMI producing additional jitter and phase noise in the oscillator and compensation electronics [133]	Differential topology with dynamic phase injection reducing EMI-induced phase noise by 10–15 dB in a high-frequency crystal oscillator circuit [133]
**OCXO**	
Resonance-frequency shifts caused by static electric fields in SC-cut resonators [13] Long-term frequency drift caused by electric-field-induced migration of mobile ionic impurities [99]	Electric and magnetic shielding and signal-path separation [27,133], alongside the minimization of dc voltages on the electrodes to prevent impurity migration within the resonator [99]
**MEMS oscillators with passive compensation**	
Magnetic-field-induced frequency shifts caused by ferromagnetic package or mounting components [13,136] Large frequency shifts in MEMS structures incorporating magnetostrictive films, with a reported sensitivity of 28.5 Hz/nT [138]	Avoidance of ferromagnetic and magnetostrictive materials in oscillator structures not intended for magnetic sensing [136,138] Package-level magnetic shielding and reduction of parasitic electromagnetic coupling [133,136]
**MEMS oscillators with active compensation (OCMO)**	
Increased susceptibility to electromagnetic coupling in highly miniaturized systems with limited shielding, grounding, and PCB-level isolation Frequency error and phase-noise degradation caused by EMI coupling into integrated bias, temperature-control, and feedback loops	Package- and board-level shielding, low-impedance grounding, supply filtering, and layout-level isolation [27,133] EMI-robust design of integrated sustaining, bias and compensation electronics

**Table 10:** Effects of ionizing radiation on quartz and MEMS oscillator stability.

Effects	Compensation and Mitigation
**XO**	
Transient increase in series resistance and reduction in *Q*, causing amplitude and phase disturbances after pulsed X-ray or gamma irradiation at doses on the order of $10^{4}$ rad [139,140] Persistent frequency shifts caused by radiation-induced modification of Al–Li/Na impurity-related defects, reaching tens of ppm in natural quartz after high accumulated doses [13]	Use of high-purity synthetic quartz to reduce radiation-induced frequency shifts [13,15] Annealing and electrodiffusion processing (sweeping) to reduce the concentration of mobile alkali ions and improve radiation tolerance [13]
**TCXO**	
Approximately linear frequency shift with total ionizing dose at low doses, exhibiting a slope change above about 40 Gy, followed by saturation and mode hopping in one of the two tested models [142]	Use of the approximately linear radiation-induced frequency shift for onboard TID monitoring, with a reported dose-estimation error of ±8% above 20 Gy for the better-behaved of the two tested oscillators and ±20% for the TCXO [142]
**OCXO**	
Transient loop-amplitude and phase disturbances during pulsed irradiation [139] Radiation-induced degradation of the oven-control and sustaining electronics	Selection of radiation-resistant quartz material, processing technology, crystal cut, and control electronics [13] Radiation hardening and shielding of the oven-control and sustaining circuits
**MEMS oscillators with passive compensation**	
Radiation-induced frequency shifts and operational degradation caused by charge trapping in dielectric layers and at material interfaces [143] Shifts in resonance frequency and bias point, changes in pull-in voltage, and possible stiction in electrostatically transduced devices with exposed dielectrics [143] Resonance-frequency decrease of approximately 25 ppm in a piezoresistive structure after 10 keV X-ray exposure to 2.1 Mrad [144] Frequency shifts associated with changes in surface charge, carrier concentration, and effective Young’s modulus [144,145] Frequency shifts of opposite sign depending on the relative contributions of ionization and displacement damage under proton irradiation [145]	Grounded polysilicon shielding over radiation-sensitive dielectric layers to reduce charge-trapping effects [143] Reduction of exposed dielectric areas and use of radiation-tolerant materials and interfaces [143] Use of alternative transduction methods or strongly shielded electrostatic architectures in high-dose environments [143]
**MEMS oscillators with active compensation (OCMO)**	
Radiation-induced degradation of integrated CMOS oven-control, sustaining, bias, and readout circuits [143] Frequency error and impaired temperature stabilization caused by radiation effects in sensors, references, and feedback loops	Radiation hardening of the readout, sustaining, bias, and temperature-control electronics [143] Shielding and radiation-tolerant design of integrated control loops and reference circuits [27,143]

## Data Availability

No new data were created or analyzed in this study. Data sharing is not applicable to this article.

## Appendix A. Quantitative Comparison of Quartz and MEMS Frequency References

Table A1 includes only sources reporting an explicit comparable value. Results are grouped by measurement family and operating parameter, then ordered within comparable conditions. The evaluation level distinguishes resonator measurements, oscillator prototypes, complete-system tests, and manufacturer data, while a dedicated column records the compensation method together with the environmental or qualification context reported for each entry. Short-term stability is reported in whichever domain the cited source provides, frequency-domain phase noise or time-domain ADEV and MDEV, as these are equivalent representations of the same underlying noise process.

**Table A1:** Comparable numerical results reported for quartz and MEMS oscillators, grouped by measurement family and operating parameter.

Value Order	Technology/Device or Approach	Evaluation Level	Reported Result	Conditions/Time Scale	Power/Warm-Up	Compensation, Environment, and Qualification	Refs.
**A. Fractional frequency deviation and static frequency stability (** $\Delta f/f_{0}$ **)**							
**Temperature**							
*Absolute reported frequency residual, normalized to ppb*							
1	Quartz OCXO, segmented-polynomial correction	Laboratory oscillator prototype	Improved from ±4.29 to ±0.153 ppb	−40 to 85 °C	—	Secondary segmented temperature correction	[41]
2	SiT7101 ruggedized MEMS OCXO	Commercial packaged product	Temperature stability options ±0.5/1/3/5 ppb	−40 to 95 °C	420 mW steady/750 mW warm-up typical; rated stability in 60 s under stated preconditioning	Ruggedized oven control; manufacturer specification only	[88]
3	SiT5811 holdover MEMS OCXO, 10–60 MHz	Commercial packaged product	Temperature ±1 ppb guaranteed; slope ±0.01 ppb/°C typical	−40 to 95 °C; aging includes 8 °C ITU-T profile and 1 m/s airflow	420 mW typical at 3.3 V	Internal thermal stabilization; product specification only	[87]
4	SiT5812 holdover MEMS OCXO, 60–220 MHz	Commercial packaged product	Temperature ±1 ppb guaranteed; slope ±0.01 ppb/°C typical	−40 to 95 °C; aging includes 8 °C ITU-T profile and 1 m/s airflow	460 mW typical at 3.3 V	Internal thermal stabilization; product specification only	[92]
5	Dual Lamé-mode ovenized MEMS	Epi-seal resonator platform with oscillator loop	±1.5 ppb during the ramp; about ±0.5 ppb for one week	−40 to 60 °C over 10 h; one week at 20 °C; ADEV at τ=1000 s	<30 mW during ramp; 12 mW at 20 °C	Dual-mode thermometry and integrated micro-oven	[20]
6	Nonlinear silicon BAW oscillator	Laboratory oscillator/resonator experiment	±1.88 ppb	1.47 °C window around the 56 °C inflection point	—	Narrow temperature and amplitude operating region	[70]
7	Miniature heater-embedded quartz OCXO	Laboratory oscillator prototype	±3 ppb	−40 to 85 °C	—	Symmetric heater-embedded ceramic package	[38]
8	SiT5543 ruggedized MEMS Super-TCXO	Commercial packaged product	Temperature stability ±5 ppb	−40 to 95 °C option	About 145 mW typical at 3.3 V; rated stability in 0.2 s typical	Electronic compensation; conditions and limits are manufacturer specified	[89]
9	Duffing-assisted MEMS reference	Resonator experiment	±14 ppb	Transient ambient-temperature changes	—	Nonlinearity-mediated drift suppression; drive-level dependent	[69]
10	Quartz OCXO in a 5×3.2 mm package	Laboratory oscillator prototype	±20 ppb	−40 to 95 °C	—	Miniaturized ovenized package	[39]
11	MEMS oscillator with two-stage off-chip oven	Laboratory oscillator	±63 ppb	Chamber at −10 °C; oscillator held near 35 °C to ±1 mK	—	Two-stage fuzzy PID thermal control; not an integrated product	[66]
12	ScAlN-on-Si dual-resonator OCMO	Resonator platform with closed control loop	±100 ppb	−40 to 85 °C	Maximum heating power 40 mW	Co-located sensing resonator and micro-oven	[62]
13	Dual-mode piezoelectric OCMO	Resonator experiment with PLL control	±190 ppb	−40 to 105 °C	Maximum 39 mW	PLL locks the operating mode to its turnover point	[63]
14	100 MHz quartz TCXO, closed correction loop	Laboratory oscillator prototype	±0.22 ppm	−40 to 85 °C	—	Closed real-time temperature correction	[35]
15	50 MHz MEMS oscillator with DFT calibration	System simulation	±0.3 ppm; frequency-estimation discrepancy <±0.4 Hz	—	—	Three-point interpolated DFT and PLL feedback; simulated result	[61]
16	Structurally sensed oven-controlled MEMS oscillator	Integrated oscillator prototype	Temperature stability ±0.3 ppm	Source-reported operating range	—	OCMO result; conditions retained in the cited source	[19]
17	Quartz TCXO, higher-order analog multiplier	Post-layout simulation	±0.8 ppm, simulated	−40 to 95 °C	—	Simulated in 0.13 μm CMOS; not a measured product result	[36]
18	48 MHz quartz TCXO, time-domain enhancement	Laboratory oscillator prototype	±1.4 ppm	−55 to 115 °C	—	Time-domain accuracy-boosting correction	[37]
19	Low-power analog quartz TCXO	Integrated oscillator prototype	Temperature stability ±2.5 ppm	−40 to 80 °C	185 μW	Conference result; temperature range and power reported together	[33]
20	Quartz TCXO with polynomial varactor correction	Integrated oscillator prototype	±3.75 ppm	−30 to 90 °C	—	Piecewise-polynomial varactor compensation	[130]
21	32.768 kHz quartz TCXO	Integrated oscillator prototype	Temperature error ±4.2 ppm	−20 to 85 °C; power quoted at room temperature	43 nW	Piecewise-linear correction by ΔΣ-modulated load capacitance	[129]
22	Mechanically coupled square-extensional MEMS	Resonator measurement, 18 MHz	Minimum shift ±10 ppm	−40 to 85 °C	—	Coupling ratio sets the effective TCF	[58]
23	Doped silicon bulk-mode capacitive MEMS	Resonator measurement	±16 ppm, square-extensional; ±20 ppm, length-extensional	−40 to 85 °C; $22.5^{\circ }$ from 〈110〉	—	Doping, crystal orientation, and modal selection	[53]
24	ScAlN-on-Si MEMS with oxide composite	Resonator measurement, 24.44 MHz	±21.5 ppm; measured $Q_{L}=10,017$	−40 to 85 °C	—	Doping, orientation, and oxide composite compensation; deduced unloaded value subsequently revised	[54,55]
25	Modal-engineered pseudo-Lamé MEMS	Resonator measurement	$TCF_{1}=-0.39$ ppm/°C; TCF2=−7.3 ppb/°C2; total deviation 38 ppm	−20 to 70 °C	—	Geometry controls modal participation	[57]
26	Coupled piezoelectric and silicon MEMS modes	Resonator measurement, 25.202 MHz	±55 ppm; $Q_{\mathrm{un}}=80,695$; average *Q* gain 2.44× and drift reduction 2.5×	−40 to 85 °C	—	Lithographically set coupling improves *Q* and TCF together	[60]
27	SilOx MEMS resonator with embedded oxide pillars	Resonator measurement	Overall drift 83 ppm; improved *Q* stability	−40 to 80 °C	—	Composite stress compensation; no reported insertion-loss penalty	[48]
28	${\mathrm{Si/SiO}}_{2}$ composite MEMS resonator	Wafer-encapsulated resonator measurement	Total frequency variation <200 ppm	−40 to 125 °C	—	Opposing silicon and oxide temperature coefficients	[46]
29	Highly doped TPoS extensional MEMS	Resonator measurement, ∼25 MHz	Total variation <245 ppm	−40 to 85 °C	—	Doping, orientation, and film-thickness tuning	[52]
30	Folded-anchor CMOS–MEMS resonator	Resonator measurement	TCF reduced by 100×; total shift 2500 ppm; turnover at 27 °C	0 to 90 °C	—	Thermally induced stresses balanced by anchor geometry	[56]
**Temperature gradients, hysteresis, thermal cycling, and self-heating**							
*Dynamic or hysteretic frequency residual, normalized to ppb*							
1	Quartz oscillator with LSTM correction	Data-driven oscillator model	RMSE <0.05 ppm	Combined temperature and hysteresis prediction	—	LSTM and transfer learning; model-dependent result	[102]
2	Quartz TCXO with trajectory-aware model	Oscillator experiment	Residual reduced from about 700 to 150 ppb	Thermal cycles over −40 to 80 °C	—	State-dependent f(T,H) compensation	[32]
*Residual fraction of the applied disturbance, in percent*							
1	Quartz crystal microbalance with spatial correction	Sensor/resonator-level experiment	Residual error reduced below 5% of the initial disturbance	Dynamic nonuniform temperature field	—	Correction uses mean temperature and spatial gradients; QCM result, not a timing oscillator	[94]
**Supply voltage, electrical load, and loop electronics**							
*Supply-induced frequency residual, normalized to ppb*							
1	Regulated industrial quartz OCXO	Complete oscillator prototype	Supply-induced error below 5 ppt	5% supply variation	—	Regulated low-noise architecture; phase-noise conditions source specific	[40]
2	Miniature quartz OCXO	Laboratory oscillator prototype	Frequency error within ±1 ppb	3.3 V supply varied by ±5%	—	Internal supply stabilization	[42]
3	Digitally controlled quartz TCXO	Integrated oscillator prototype	Frequency error ±0.16 ppm for ±10% change in $V_{DD}$	Supply-variation test	—	Analog amplitude-control loop	[130]
**Ionizing radiation**							
*Absolute reported frequency residual, normalized to ppb*							
1	Piezoresistive MEMS cantilever	Sensor/resonator irradiation experiment	Resonance frequency decreased by about 25 ppm	10 keV X-rays; accumulated dose 2.1 Mrad	—	Surface charge, carrier concentration, and modulus changes; not a complete timing oscillator	[144]
**B. Allan deviation (ADEV)**							
**Measurement framework and sensor-system context**							
*ADEV magnitude*							
1	SiT7101 ruggedized MEMS OCXO	Commercial packaged product	ADEV $4.5\times 10^{-12}$	Manufacturer-specified typical values at τ=1, 10, and 100 s	420 mW steady/750 mW warm-up typical; rated stability in 60 s under stated preconditioning	Ruggedized oven control; manufacturer specification only	[88]
2	SiT5543 ruggedized MEMS Super-TCXO	Commercial packaged product	ADEV $1.5\times 10^{-11}$ at 10 s	Manufacturer-specified value at τ=10 s	About 145 mW typical at 3.3 V; rated stability in 0.2 s typical	Electronic compensation; conditions and limits are manufacturer specified	[89]
3	Nodal-anchor silicon BAW MEMS	Laboratory resonator oscillator	$Q=1.8\times 10^{6}$ at 1.25 MHz; ADEV 36 ppt at 0.4 s; drift 0.3 ppm at 2 h	No active thermal compensation	—	Excellent short-term result but ambient temperature dominates later	[79]
4	Honeycomb phononic-crystal OCMO MEMS	Micro-oven resonator platform	$Q_{\mathrm{un}}=42,784$; ADEV 0.62 ppb	τ=1.02 s under micro-oven control	—	Phononic lattice suppresses anchor loss	[80]
5	Five AlN-on-Si MEMS oscillators	Matched multimode oscillator experiment	Q=18,600, ADEV $5.5\times 10^{-9}$ at 1 s; ADEV $4\times 10^{-9}$ from Q=4230 mode	τ=1 s; five modes of one resonator	—	Shows that maximum *Q* does not alone guarantee best oscillator stability	[81]
**Temperature**							
*ADEV magnitude*							
1	Dual Lamé-mode ovenized MEMS	Epi-seal resonator platform with oscillator loop	Minimum ADEV <0.65 ppb	τ=1000 s; dual-mode oven control	<30 mW during ramp; 12 mW at 20 °C	Dual-mode thermometry and integrated micro-oven	[20]
2	Blue-sideband-excited multifunctional MEMS	Resonator experiment	ADEV 1.5 ppb; 11.9 ppb with combined thermal and electrostatic disturbance	τ=1000 s	—	Sum-frequency reference; prescribed modal ratio and simultaneous sensing	[74]
3	Curved-beam piezoelectric MEMS	Resonator measurement	ADEV 127 ppb during ramp; 2.0 ppb near insensitive extremum	τ=1.45 s over 25–75 °C; τ=2.51 s near 55 °C	—	Beat-frequency processing or selection of a temperature-insensitive mode	[90]
**Temperature gradients, hysteresis, thermal cycling, and self-heating**							
*ADEV magnitude*							
1	Quartz OCXO thermal model and experiment	Complete oscillator/system study	ADEV of order $10^{-13}$ at 100 s required ambient fluctuations below 10 mK	Short-term stability under ambient-temperature fluctuation	—	Couples resonator thermal response with the oven loop	[96]
2	Ultrastable quartz oscillators in controlled chamber	Environmental-chamber system test	Stability improved about 100× to $2\times 10^{-13}$	τ=1 to $10^{5}$ s; chamber held to ±25 mK, ±0.1% RH, and ±12 Pa	—	Simultaneous temperature, humidity, and pressure stabilization	[97]
**C. Modified Allan deviation (MDEV)**							
**Temperature**							
*MDEV magnitudes*							
1	268 MHz DFRT micromechanical reference	Prototype clock with encapsulated resonator	MDEV $∼8\times 10^{-12}$ at 8 h	τ=8 h; 48 h laboratory record near the 65 °C turnover	—	Mode-ratio thermometry and amplitude-based FLL; prototype demonstration	[67]
2	Single-mode Lamé MEMS reference	Resonator plus sustaining-loop experiment	MDEV 42 ppt at 85 s; sub-ppb to 4800 s	Constant-temperature laboratory test	—	Laboratory thermal isolation; not an integrated product	[68]
**D. Resonator quality factor and** fQ **product**							
**Measurement framework and sensor-system context**							
*fQ product or physical limit*							
1	Quartz, silicon, and AlN resonators	Material-level prediction below 1 GHz	Quartz: 1.8–$3.2\times 10^{13}$ Hz; silicon: 2.5–$3.9\times 10^{13}$ Hz; AlN: 0.9–$2.5\times 10^{13}$ Hz	Phonon-interaction limit; not device ADEV	—	Physical limit, not demonstrated oscillator performance	[17]
**Temperature**							
*Measured quality factor Q*							
1	Nodal-anchor silicon BAW MEMS	Laboratory resonator oscillator	Measured $Q=1.8\times 10^{6}$	No active thermal compensation	—	Excellent short-term result but ambient temperature dominates later	[79]
2	Coupled piezoelectric and silicon MEMS modes	Resonator measurement, 25.202 MHz	Measured $Q_{\mathrm{un}}=80,695$	−40 to 85 °C	—	Lithographically set coupling improves *Q* and TCF together	[60]
3	Honeycomb phononic-crystal OCMO MEMS	Micro-oven resonator platform	Measured $Q_{\mathrm{un}}=42,784$	τ=1.02 s under micro-oven control	—	Phononic lattice suppresses anchor loss	[80]
4	Five AlN-on-Si MEMS oscillators	Matched multimode oscillator experiment	Measured Q=18,600 among five modes	τ=1 s; five modes of one resonator	—	Shows that maximum *Q* does not alone guarantee best oscillator stability	[81]
5	ScAlN-on-Si MEMS with oxide composite	Resonator measurement, 24.44 MHz	Measured $Q_{L}=10,017$	−40 to 85 °C	—	Doping, orientation, and oxide composite compensation; deduced unloaded value subsequently revised	[54,55]
**E. Aging, drift, retrace, and holdover**							
**Temperature gradients, hysteresis, thermal cycling, and self-heating**							
*Quoted or descriptively normalized drift magnitude, as reported (units vary by source)*							
1	268 MHz DFRT micromechanical reference	Prototype clock with encapsulated resonator	48 h linear-fit drift −4.1±2.5 ppt/day	48 h laboratory record near the 65 °C turnover	—	Mode-ratio thermometry and amplitude-based FLL; prototype demonstration	[67]
2	Characterized ${\mathrm{Si/SiO}}_{2}$ composite MEMS resonator	Resonator measurement	Drift <2 ppm over a record longer than 6 months	Turnover tunable from −55 to 125 °C; record >6 months	—	Composite compensation; dielectric charging remains a caveat	[47]
*Retrace magnitude, in ppb*							
1	Dual-mode digital quartz OCXO	Complete oscillator experiment	Recovery to within ±0.2 ppb of the pre-shutdown frequency after about 10 h	Power-off, cool-down, and restart	Oven; approximately 10 h recovery	Dual-mode excitation; value is retrace, not initial warm-up	[101]
*Holdover time error, in microseconds*							
1	ROD2522S2 quartz OCXO	Complete oscillator/system test	Time error <1.5 μs over 24 h; hysteresis $<\pm 5\times 10^{-11}$	40 °C cycle at 1 °C/min after 52 h learning	—	GNSS-disciplined aging learning plus thermomechanical optimization	[95]
**Aging and long-term drift**							
*Quoted or descriptively normalized drift magnitude, in ppt/day*							
1	Quartz OCXO with resonator-pin phase monitoring	Complete oscillator experiment	Aging rate $9.8\times 10^{-12}$/day after compensation	Long-term drift monitoring	Oven; added phase-monitoring electronics	Reference-free compensation from phase shift across resonator pins	[107]
2	SiT7101 ruggedized MEMS OCXO	Commercial packaged product	Aging ±80 ppb over 20 years at 50 °C	Manufacturer long-term rating at 50 °C	420 mW steady/750 mW warm-up typical; rated stability in 60 s under stated preconditioning	Ruggedized oven control; manufacturer specification only	[88]
3	SiT5543 ruggedized MEMS Super-TCXO	Commercial packaged product	20-year aging magnitude 150 ppb maximum	Manufacturer long-term rating	About 145 mW typical at 3.3 V; rated stability in 0.2 s typical	Electronic compensation; conditions and limits are manufacturer specified	[89]
4	SiT5811 holdover MEMS OCXO, 10–60 MHz	Commercial packaged product	Aging ±0.1 ppb/day	Includes an 8 °C ITU-T profile and 1 m/s airflow	420 mW typical at 3.3 V	Internal thermal stabilization; product specification only	[87]
5	SiT5812 holdover MEMS OCXO, 60–220 MHz	Commercial packaged product	Aging ±0.1 ppb/day	Includes an 8 °C ITU-T profile and 1 m/s airflow	460 mW typical at 3.3 V	Internal thermal stabilization; product specification only	[92]
6	Commercial quartz and MEMS samples	Matched comparative laboratory test	Quartz: 0.02–0.25 ppm downward drift; MEMS: 0.19–2.83 ppm	Common test system for 2928 h; sample set	—	Direct comparison, but limited devices and not representative of current products	[109]
7	Wafer-level vacuum-encapsulated silicon MEMS	Resonator comparison	BAW drift <0.1 ppm/month; flexural devices 4 to >500 ppm/month	Same encapsulation process; device-dependent anchors/modes	—	Package stress and anchoring dominate the spread	[86]
**F. Environmental sensitivity coefficients and qualification thresholds**							
**Temperature**							
*First-order temperature coefficient magnitude, as reported (units vary by source)*							
1	Characterized ${\mathrm{Si/SiO}}_{2}$ composite MEMS resonator	Resonator measurement	$TCF_{1}=0$; TCF2≈−20 ppb/°C2	Turnover tunable from −55 to 125 °C; record >6 months	—	Composite compensation; dielectric charging remains a caveat	[47]
2	Degenerately doped silicon MEMS	Material characterization and resonator arrays	$TCF_{1}$ of $c_{11}-c_{12}$ crosses zero near $2\times 10^{19}$ cm^−3^	Measurements over −40 to 85 °C	—	Doping-, mode-, and orientation-dependent design rules; no single device-level bound	[50,51]
3	Modal-engineered pseudo-Lamé MEMS	Resonator measurement	$TCF_{1}=-0.39$ ppm/°C; TCF2=−7.3 ppb/°C2; total deviation 38 ppm	−20 to 70 °C	—	Geometry controls modal participation	[57]
4	Micro-oven MEMS in wafer-level vacuum package	Packaged resonator platform	TCF≈0.6 ppm/°C	−40 to 80 °C	—	Thermal isolation and package-stress reduction	[65]
**Acceleration, vibration, shock, and mounting stress**							
*Acceleration sensitivity, in ppb/g*							
1	SiT5543 ruggedized MEMS Super-TCXO	Commercial packaged product	Acceleration sensitivity 0.01 ppb/g maximum, grade B	Manufacturer-specified ruggedized product grade	About 145 mW typical at 3.3 V; rated stability in 0.2 s typical	Electronic compensation; conditions and limits are manufacturer specified	[89]
2	SiT7101 ruggedized MEMS OCXO	Commercial packaged product	Acceleration sensitivity 0.01 typical/0.015 maximum ppb/g; 20,000 g shock	Shock qualification per MIL-STD-883F	420 mW steady/750 mW warm-up typical; rated stability in 60 s under stated preconditioning	Ruggedized oven control; manufacturer specification only	[88]
3	Paired AT-strip quartz oscillators	Laboratory oscillator/resonator test	<0.1 ppb/g	10–40 MHz	—	Opposed acceleration-sensitivity vectors	[111]
4	Third-overtone quartz oscillator	Laboratory oscillator test	Approximately 0.3 ppb/g	Vibration excitation 200–2000 Hz	—	Selective third-overtone operation	[115]
5	Electrode-optimized quartz oscillator	Commercial-type oscillator design/test	Approximately 0.5 ppb/g	25 MHz; axis and setup source specific	—	Electrode region shifted relative to anchors	[114]
6	Centrally anchored Lamé-mode MEMS oscillator	Laboratory oscillator/resonator test	2.1 ppb/g in-plane; 3.8 ppb/g out-of-plane; central anchor 0.74 ppb/g; transient −7.7 ppm at 1.7 ms	One 11,000 g impact; operation retained after eight 5000 g and four 11,000 g shocks	—	Separates sensitivity, transient error, and survival	[117]
7	Miniature AT-cut quartz resonator/XO class	Generic resonator/oscillator class	Typical sensitivity about 2 ppb/g	Small-signal, axis-dependent response	—	Baseline value; mounting, cut, and orientation dependent	[15,27,118]
**Supply voltage, electrical load, and loop electronics**							
*Supply-voltage sensitivity, in ppb/V*							
1	High-quality quartz oscillator class	Generic complete oscillator	Representative sensitivity $5\times 10^{-11}$/V	Architecture- and condition-dependent	—	Low-noise regulation, filtering, and drive-level control	[13,15]
**Electric and magnetic fields and electromagnetic interference**							
*Magnetic sensitivities*							
1	Quartz oscillators for satellite instrumentation	Complete oscillator measurements	Resonator sensitivity of order 1 ppt/G	Orientation and construction dependent	—	Nonmagnetic supports and magnetic shielding	[137]
**Ionizing radiation**							
*Dose-estimation error, in percent*							
1	Commercial quartz oscillator/TCXO modules	Complete product irradiation test	Slope change above about 40 Gy; dose-estimation error ±8% above 20 Gy for the better unit and ±20% for the TCXO	Total-ionizing-dose exposure; two tested models	—	Frequency shift used as a dosimeter; one model saturated and mode hopped	[142]
**G. Power consumption and warm-up**							
**Temperature**							
*Typical or steady-state power, normalized to mW*							
1	32.768 kHz quartz TCXO	Integrated oscillator prototype	43 nW at room temperature; temperature error ±4.2 ppm	−20 to 85 °C; power quoted at room temperature	43 nW	Piecewise-linear correction by ΔΣ-modulated load capacitance	[129]
2	Low-power analog quartz TCXO	Integrated oscillator prototype	185 μW; temperature stability ±2.5 ppm	−40 to 80 °C	185 μW	Power ordered separately from the temperature residual	[33]
3	Dual Lamé-mode ovenized MEMS	Epi-seal resonator platform with oscillator loop	<30 mW during the ramp; 12 mW at 20 °C	−40 to 60 °C over 10 h; one week at 20 °C; ADEV at τ=1000 s	<30 mW during ramp; 12 mW at 20 °C	Dual-mode thermometry and integrated micro-oven	[20]
4	Dual-mode piezoelectric OCMO	Resonator experiment with PLL control	Maximum heating power 39 mW	−40 to 105 °C	Maximum 39 mW	PLL locks the operating mode to its turnover point	[63]
5	ScAlN-on-Si dual-resonator OCMO	Resonator platform with closed control loop	Maximum heating power 40 mW	−40 to 85 °C	Maximum heating power 40 mW	Co-located sensing resonator and micro-oven	[62]
6	SiT5543 ruggedized MEMS Super-TCXO	Commercial packaged product	About 145 mW typical at 3.3 V	−40 to 95 °C option; 19.2 MHz, no load for power value	About 145 mW typical at 3.3 V; rated stability in 0.2 s typical	Electronic compensation; conditions and limits are manufacturer specified	[89]
7	SiT5811 holdover MEMS OCXO, 10–60 MHz	Commercial packaged product	420 mW typical at 3.3 V	−40 to 95 °C; aging includes 8 °C ITU-T profile and 1 m/s airflow	420 mW typical at 3.3 V	Internal thermal stabilization; product specification only	[87]
8	SiT7101 ruggedized MEMS OCXO	Commercial packaged product	420 mW steady; 750 mW warm-up typical	−40 to 95 °C; ADEV typical at τ=1, 10, and 100 s; shock per MIL-STD-883F	420 mW steady/750 mW warm-up typical; rated stability in 60 s under stated preconditioning	Ruggedized oven control; manufacturer specification only	[88]
9	SiT5812 holdover MEMS OCXO, 60–220 MHz	Commercial packaged product	460 mW typical at 3.3 V	−40 to 95 °C; aging includes 8 °C ITU-T profile and 1 m/s airflow	460 mW typical at 3.3 V	Internal thermal stabilization; product specification only	[92]
**Start-up and power cycling**							
*Warm-up or time-to-rated-stability, in seconds*							
1	SiT5543 ruggedized MEMS Super-TCXO	Commercial packaged product	Rated stability in 0.2 s typical	−40 to 95 °C option; 19.2 MHz, no load for power value	About 145 mW typical at 3.3 V; rated stability in 0.2 s typical	Electronic compensation; conditions and limits are manufacturer specified	[89]
2	Quartz OCXO with external thermoelectric cooler	Commercial OCXO on a test PCB	Warm-up reduced from about 3 min to <1 min	Start-up transient	Added TEC and thermistor-control module	External active cooling/heating adds volume and power	[44]
3	SiT7101 ruggedized MEMS OCXO	Commercial packaged product	Rated stability in 60 s under stated preconditioning	−40 to 95 °C; ADEV typical at τ=1, 10, and 100 s; shock per MIL-STD-883F	420 mW steady/750 mW warm-up typical; rated stability in 60 s under stated preconditioning	Ruggedized oven control; manufacturer specification only	[88]
